# KDM6B safeguards mineralized tissue homeostasis from mechanical stress through epigenetic control of PIEZO1-mediated mechanotransduction in the mouse incisor

**DOI:** 10.1038/s41413-026-00544-2

**Published:** 2026-05-28

**Authors:** Lin Meng, Mingyi Zhang, Jifan Feng, Tingwei Guo, Hana Hekmat, Heliya Ziaei, Peng Chen, Aaron Harouni, Thach-Vu Ho, Yang Chai

**Affiliations:** Center for Craniofacial Molecular Biology, Herman Ostrow School of Dentistry, University of Southern California, Los Angeles, CA USA

**Keywords:** Bone quality and biomechanics, Calcium and vitamin D

## Abstract

Mechanical forces shape the growth and regeneration of mineralized tissues such as bones and teeth, yet how these tissues adapt to sustained mechanical stress remains poorly understood. Here, using mouse incisor models with varying degrees of loading, we identified that the histone demethylase KDM6B is a critical epigenetic regulator that preserves mineralized tissue homeostasis by protecting progenitor transit-amplifying cells from mechanical stress-induced apoptosis. Loss of *Kdm6b* impairs this balance by enhancing PIEZO1-dependent mechanotransduction, leading to excessive Ca^2+^ influx and apoptosis in transit-amplifying cells. Mechanistically, *Kdm6b* deficiency increases H3K27me3 at the *Bmi1* promoter, silencing its expression and derepressing *Piezo1* expression. Importantly, *Piezo1* haploinsufficiency in *Kdm6b*-deficient mice restores Ca^2+^ influx restriction, rescuing transit-amplifying cell defects and tissue homeostasis. These findings reveal that KDM6B-H3K27me3-BMI1-PIEZO1 is a critical epigenetic “mechanostat” that protects dental progenitor cells from mechanical stress, ensuring sustained tissue homeostasis. This chromatin-based mechanism of tissue mechano-adaptation could be targeted to prevent mechanically induced degeneration in mineralized tissues.

## Introduction

Tissue homeostasis is a dynamic process that preserves the structural and functional integrity of tissues in multicellular organisms despite continuous exposure to fluctuating environmental conditions. This equilibrium is sustained through reciprocal interactions between cells and their specialized microenvironments.^1^ This microenvironment is modulated by a diverse array of extracellular cues.^2,3^ Among these stimuli, mechanical forces are particularly critical for maintaining homeostasis in mineralized tissues such as bones and teeth, which bear the brunt of activities like walking and chewing.^4–6^ Mechanical loading is well known to be essential for preserving skeletal mass and quality in humans and other animals.^7,8^ Loss of mechanical input results in rapid bone loss^9^; On the other hand, excessive mechanical stress contributes to degenerative conditions such as osteoarthritis.^10,11^ Thus, understanding how mineralized tissues sustain homeostasis under mechanical stress is a fundamental question in skeletal biology.

Through mechanotransduction, cells detect physical forces, such as tension, compression, and shear stress, and convert them into biochemical signals that govern cell behavior.^12–14^ However, not all mechanical forces elicit proportional biological responses. Recent studies suggest that intestinal stem cells are exposed to forces ranging from 0 to 25 kPa, but their responsiveness peaks at approximately 12 kPa.^15^ This non-linear behavior suggests that intrinsic regulatory mechanisms fine-tune or buffer mechanical signaling to prevent cellular damage under excessive loading. The molecular basis of this protective adaptation, particularly in mineralized tissues, remains largely unexplored.

Epigenetic modifications regulate gene expression independently of alterations to the DNA sequence.^16^ They represent a critical regulatory layer governing cell fate specification during embryogenesis and tissue homeostasis throughout an organism’s lifespan.^17–20^ Histone H3 lysine 27 trimethylation (H3K27me3), a repressive chromatin mark, is implicated in cellular responses to environmental stress across species,^21,22^ suggesting that it may be an epigenetic regulator of tissue mechanoadaptation. This mark is dynamically regulated by the antagonistic actions of the methyltransferases EZH1/2 and the demethylases KDM6A, KDM6B (JMJD3), and KDM6C (UTY). Among these, KDM6B has emerged as a pivotal regulator of gene networks controlling organogenesis, bone formation, dental cementogenesis, and lineage specification.^17,23–25^ Furthermore, it is required for osteogenesis of the periodontal ligament under orthodontic force.^26^ These lines of evidence converge to suggest that KDM6B may regulate adaptive responses in mechanically stressed mineralized tissues. Elucidating this mechanism could provide critical insights into how epigenetic regulation intersects with mechanotransduction to maintain tissue integrity and yield new strategies for fighting degenerative skeletal diseases.

GLI1^+^ mesenchymal stem cells (MSCs) are essential for craniofacial bone and tooth development and repair.^27,28^ Mouse incisors grow continuously throughout life, maintained by GLI1^+^ MSCs located in the cervical loop niche. These stem cells generate transit-amplifying cells (TACs) that further differentiate into odontoblasts and pulp cells, ensuring lifelong renewal.^29^ Notably, the mouse incisor experiences significantly higher mechanical forces than other mineralized tissues.^13^ During gnawing, pressure of up to ~280 MPa is exerted at the incisor tip, whereas chewing food generates forces of ~100 MPa at the proximal end.^30^ The highly dynamic and mechanically responsive nature of the incisor provides a powerful system for exploring the molecular and epigenetic regulation of force-dependent tissue homeostasis.

In this study, we identify KDM6B as an epigenetic regulator that safeguards mineralized tissue homeostasis under mechanical stress by modulating PIEZO1-dependent mechanotransduction. Using mouse incisor models experiencing different mechanical loads, we found that loss of *Kdm6b* in the incisor GLI1^+^ lineage (*Gli1-CreER*^*T2*^*;* *Kdm6b*^*fl/fl*^ mice) induces TAC apoptosis and disrupts tissue architecture specifically under high mechanical loading through an H3K27me3-BMI1-PIEZO1 axis. Our study reveals that this previously unrecognized axis fine-tunes PIEZO1-dependent mechanotransduction to prevent force-induced TAC exhaustion and preserve mineralized tissue homeostasis. More broadly, our study reveals a new paradigm in which epigenetic regulators function as intrinsic modulators of mechanical sensitivity. These findings suggest potential biomarkers such as KDM6B and H3K27me3 to predict regenerative outcomes under physiological forces and open new therapeutic avenues for treating mechanical overloading-induced diseases such as osteoarthritis.

## Results

### KDM6B is essential for tissue homeostasis and incisor growth under mechanical loading in adult mice

**Fig. 1:**
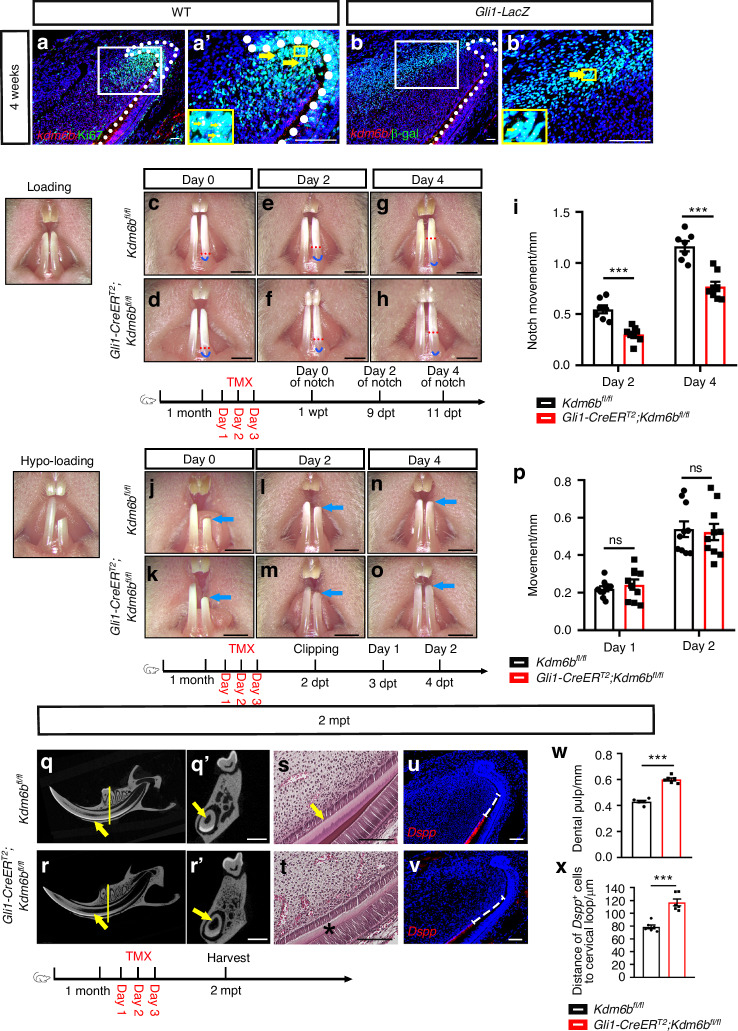
KDM6B is essential for tissue homeostasis and incisor growth under mechanical loading in adult mice. **a**, **b** *Kdm6b* in situ hybridization (red) combined with Ki67 immunofluorescence (green) in wild-type incisors (**a**) and β-galactosidase immunofluorescence (green) in *Gli1-LacZ* incisors (**b**). **a’**, **b’** Magnified views of boxed regions. White dotted lines outline the cervical loop. Yellow arrows indicate double-positive cells. Scale bars, 50 μm. **c**–**h** Representative images of notch movement under normal mechanical loading in control and *Gli1-CreER*^*T2*^*;Kdm6b*^*fl/fl*^ mice at the indicated time points. Red dotted lines indicate notch position and blue lines mark the gingival margin. Scale bars, 1 mm. **i** Quantification of notch movement on day 2 and day 4 (mean ± SEM, *n* = 7; day 2, *P* = 0.000 1; day 4, *P* < 0.000 1). **j–o** Representative images of clipped incisors under hypo-loading in control and *Gli1-CreER*^*T2*^*;Kdm6b*^*fl/fl*^ mice. Blue arrows indicate clipping position. Scale bars, 1 mm. **p** Quantification of incisor growth on day 1 and day 2 (mean ± SEM, *n* = 10; day 1, *P* = 0.475 7; day 2, *P* = 0.805 9). **q–r** Micro-CT images of control and *Gli1-CreER*^*T2*^*;Kdm6b*^*fl/fl*^ incisors at 2 mpt with coronal sections through the first molar furcation (**q’**, **r’**). Yellow arrows indicate dental pulp. Scale bars, 1 mm. **s**, **t** H&E staining showing dentin defects (asterisk). **u**, **v** *Dspp* in situ hybridization (red). White dotted lines indicate the distance between the cervical loop and odontoblast initiation site. Scale bars, 50 μm. **w**, **x** Quantification of pulp cavity diameter and the distance between *Dspp*^+^ cells and the cervical loop (mean ± SEM, *n* = 6; *P* < 0.000 1). TMX, tamoxifen; dpt, days post-tamoxifen injection; mpt, months post-tamoxifen

To study the functional roles of the KDM6 gene family, we first investigated their expression patterns by analyzing single-cell RNA sequencing (scRNA-seq) data from previous studies^31^ and performing in situ hybridization analysis. We found that *Kdm6b* exhibited the most extensive and highest level of expression among all KDM6 family members in both the mesenchymal and epithelial regions of the incisor (Fig. S1). To further elucidate the relationship between *Kdm6b*^+^ cells and GLI1^+^ MSCs, as well as *Kdm6b*^+^ cells and TACs, we performed colocalization staining of *Kdm6b*, MSCs, and TACs in *Gli1-LacZ* mice using β-gal to label GLI1^+^ MSCs and KI67 to label TACs. The results showed a significant overlap between *Kdm6b*^+^ cells and TACs (Fig. 1a, b), suggesting a potential regulatory role for *Kdm6b* in tissue homeostasis within the mouse incisor.

To determine the functional importance of *Kdm6b* under mechanical loading, we generated *Gli1-CreER*^*T2*^*;Kdm6b*^*fl/fl*^ mice to conditionally delete *Kdm6b* in the GLI1^+^ lineage following tamoxifen induction. We confirmed efficient deletion of *Kdm6b* in both epithelial and mesenchymal compartments of the incisor following induction at one month of age (Fig. S2a–c). We then assessed incisor growth by marking the labial surface near the gingival margin and tracking the movement of the notch over time. Two and four days after notching, *Gli1-CreER*^*T2*^*;Kdm6b*^*fl/fl*^ mice showed significantly reduced incisor growth compared to controls (Fig. 1c–i). Additionally, to determine whether this phenotype is dependent on mechanical stress, we established a reduced loading model by clipping a portion of the left mandibular incisor. In this hypo-loading model, the right incisor experienced normal mechanical loading, while the left was subjected to reduced loading from chewing and gnawing. Under these conditions, the growth rate on the hypo-loaded (left) side was not significantly different between control and *Gli1-CreER*^*T2*^*;Kdm6b*^*fl/fl*^ mice (Fig. 1j–p), indicating that loss of *Kdm6b* disrupts incisor growth specifically under mechanical loading conditions. To rule out the possibility that tissue injury from clipping confounded these results, we employed a bilateral clipping model that reduced gnawing-associated loading while preserving chewing forces. In this model, we observed similar growth between groups at 1 day post-trimming, but by 2 days post-trimming, incisor growth was significantly impaired in *Gli1-CreER*^*T2*^*;Kdm6b*^*fl/fl*^ mice (Fig. S2d–k). These data suggest that *Kdm6b* is required for maintaining tissue homeostasis and function specifically under mechanical loading.

To further evaluate the long-term effects of *Kdm6b* deletion under force, we performed microCT analysis at two months post-tamoxifen induction (pt). The results revealed pathological expansion of the dental pulp cavity in *Gli1-CreER*^*T2*^*;Kdm6b*^*fl/fl*^ mice (Fig. 1q, r, w). Histological analysis demonstrated reduced dentin and enamel thickness in *Gli1-CreER*^*T2*^*;Kdm6b*^*fl/fl*^ mice compared to controls (Fig. 1s, t), suggesting impaired tissue homeostasis in the adult mouse incisors. We also examined the expression of *Dspp*, a marker of terminally differentiated odontoblasts, and found that the distance between *Dspp*^+^ odontoblasts and the cervical loop was significantly increased in *Gli1-CreER*^*T2*^*;Kdm6b*^*fl/fl*^ mice (Fig. 1u, v, x), indicating compromised odontoblast differentiation. Since *Kdm6b* is also expressed in dental epithelial cells, we generated *Sox2-CreER*^*T2*^*;Kdm6b*^*fl/fl*^ mice to investigate whether the phenotypic defects resulted from the loss of epithelial *Kdm6b* alone. Notably, we did not observe similar mesenchymal defects in *Sox2-CreER*^*T2*^*;Kdm6b*^*fl/fl*^ mice, suggesting that the disruptions in tissue homeostasis were primarily driven by lack of *Kdm6b* in the mesenchymal GLI1^+^ lineage (Fig. S2l–q). Together, these findings demonstrate that *Kdm6b* is required for maintaining mineralized tissue homeostasis in response to mechanical loading, supporting its role as a key regulator of proper tissue renewal under mechanical stress.

### KDM6B maintains TAC fate under mechanical loading

The continuous growth of mouse incisors relies on coordinated proliferation and differentiation of MSCs. Previous studies have shown that GLI1^+^ MSCs first expand into TACs, which then differentiate into odontoblasts and dental pulp cells.^31^ To elucidate the cellular mechanisms underlying defective dentin formation and impaired incisor growth in *Gli1-CreER*^*T2*^*;Kdm6b*^*fl/fl*^ mice under mechanical loading, we first assessed whether the GLI^1+^ MSC population was altered following *Kdm6b* deletion. To quantify GLI1^+^ MSCs, we generated *Gli1-CreER*^*T2*^*;Kdm6b*^*fl/fl*^*;Gli1-LacZ* mice to compare with *Gli1-LacZ* control mice. At 1 wpt and 2 wpt, the number of GLI1^+^ MSCs remained unchanged in the incisor of *Gli1-CreER*^*T2*^*;Kdm6b*^*fl/fl*^*;Gli1-LacZ* mice compared to controls (Fig. S3a–f). We further examined long-term label-retaining cells, a subset of quiescent MSCs residing in the proximal incisor. To label retaining cells, we administered 5-Ethynyl-2’-deoxyuridine (EdU) daily from postnatal day 5 to one month, followed by an eight-week chase period. The number of retaining cells was significantly reduced in *Gli1-CreER*^*T2*^*;Kdm6b*^*fl/fl*^ mice compared to controls (Fig. S3g–i), suggesting that loss of *Kdm6b* impairs long-term MSC quiescence.

**Fig. 2:**
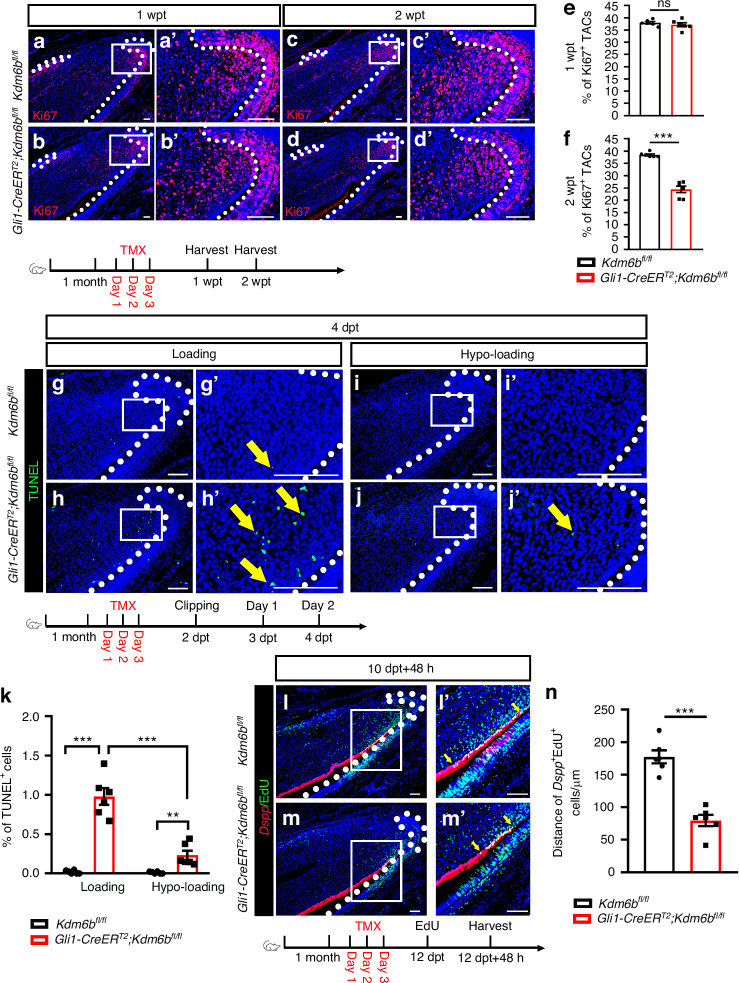
KDM6B maintains TAC fate under mechanical loading. **a–d** Ki67 immunofluorescence in control and *Gli1-CreER*^*T2*^*;Kdm6b*^*fl/fl*^ incisors at 1 wpt (**a**, **b**) and 2 wpt (**c**, **d**). **a’–d’** Magnified views of boxed regions. White dotted lines outline the cervical loop. Scale bars, 50 μm. **e, f** Quantification of Ki67^+^ cells in dental mesenchyme at 1 and 2 wpt (mean ± SEM, *n* = 6; 1 wpt, *P* = 0.396 0; 2 wpt, *P* < 0.000 1). **g–k** TUNEL staining of incisors from normally loaded (**g**, **h**) and hypo-loaded (**i**, **j**) incisors of control and *Gli1-CreER*^*T2*^*;Kdm6b*^*fl/fl*^ mice at 4 dpt. **g’–j’** Magnified views of boxed regions. White dotted lines outline the cervical loop. Yellow arrows indicate TUNEL^+^ cells. Scale bars, 50 μm. **k** Quantification of TUNEL^+^ cells in dental mesenchyme (mean ± SEM, *n* = 6; loading, *P* < 0.000 1; hypo-loading, *P* = 0.003 5; loading vs. hypo-loading, *P* < 0.000 1). **l**, **m** *Dspp* in situ hybridization (red) combined with EdU labeling (green) in control and *Gli1-CreER*^*T2*^*;Kdm6b*^*fl/fl*^ incisors. **l’**, **m’** Magnified views of boxed regions. White dotted lines outline the cervical loop. The distance between yellow arrows indicates migration distance of differentiated TACs. Scale bars, 50 μm. **n** Quantification of the distance of *Dspp*^+^EdU^+^ cells (mean ± SEM, *n* = 6; *P* < 0.000 1). wpt weeks post-tamoxifen injection

Next, we examined the TAC population after tamoxifen induction using Ki67 immunostaining. While the number of TACs remained unchanged at 1 wpt, a significant reduction was observed by 2 wpt in *Gli1-CreER*^*T2*^*;Kdm6b*^*fl/fl*^ mice (Fig. 2a–f), indicating progressive TAC depletion. To identify the underlying cause of TAC reduction, we assessed apoptosis in *Gli1-CreER*^*T2*^*;Kdm6b*^*fl/fl*^ and control mice using TUNEL staining. A significant increase in apoptosis occurred within the TAC region as early as 4 dpt in incisors of *Gli1-CreER*^*T2*^*;Kdm6b*^*fl/fl*^ mice compared to controls in mice with normal occlusion as well as in the bilateral clipping model (Fig. 2g–k and Fig. S3j–l). Notably, hypo-loaded incisors (after unilateral clipping) exhibited reduced apoptosis in *Gli1-CreER*^*T2*^*;Kdm6b*^*fl/fl*^ mice compared to normally loaded counterparts (Fig. 2g–k), indicating that *Kdm6b* is essential for TAC survival specifically under mechanical stress. To examine TAC-to-odontoblast differentiation, we performed EdU pulse labeling and analyzed the overlap of EdU⁺ cells with *Dspp*^+^ odontoblasts 48 hours later. In *Gli1-CreER*^*T2*^*;Kdm6b*^*fl/fl*^ mice, the spatial overlap between EdU^+^ and *Dspp*^+^ cells was significantly reduced at 2 wpt (Fig. 2l–n), suggesting impaired TAC differentiation into odontoblasts. Collectively, these findings demonstrate that *Kdm6b* deletion leads to apoptosis of TACs under mechanical loading, resulting in reduced TAC proliferation and differentiation, which secondarily impacts stem cell quiescence. This supports the notion that KDM6B safeguards TAC fate under mechanical stress, preserving the regenerative capacity of the MSC compartment for lifelong incisor renewal.

### Loss of *Kdm6b* leads to apoptosis of TACs via PIEZO1-mediated calcium ion hyperactivity under mechanical load

**Fig. 3:**
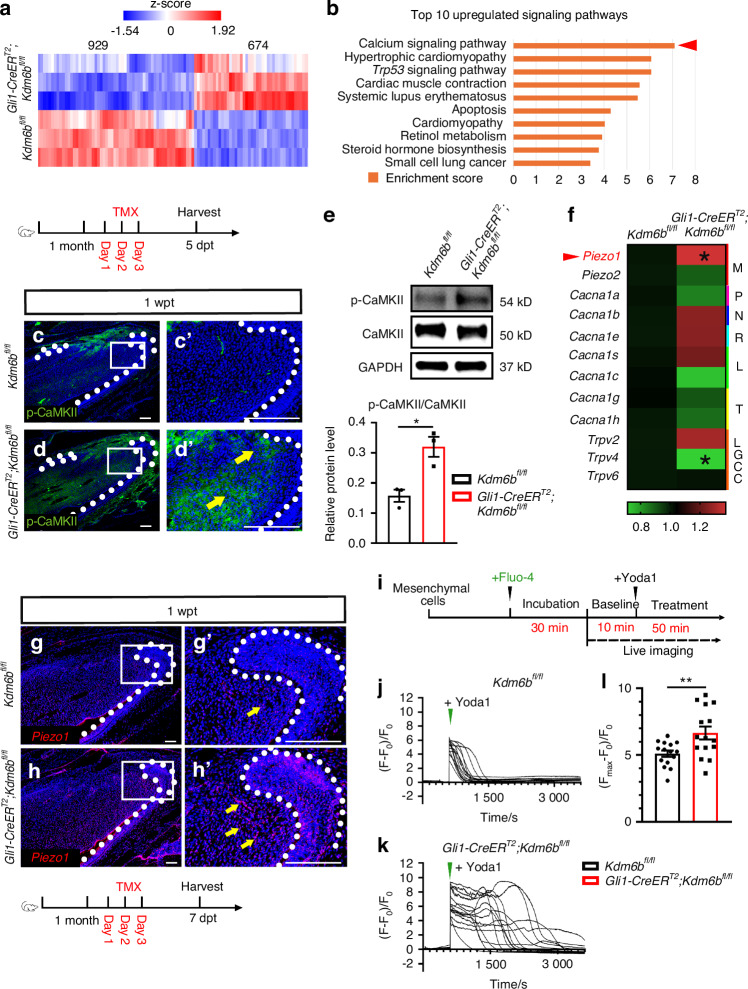
Loss of *Kdm6b* leads to apoptosis of TACs via PIEZO1-mediated calcium hyperactivity under mechanical load. **a** Heatmap of bulk RNA-seq data from the proximal region of control and *Gli1-CreER*^*T2*^*;Kdm6b*^*fl/fl*^ mice at 5 dpt. **b** Top 10 upregulated signaling pathways identified by KEGG analysis of differentially expressed genes. **c**, **d** Immunostaining of p-CaMKII in control and *Gli1-CreER*^*T2*^*;Kdm6b*^*fl/fl*^ incisors. **c’**, **d’** Magnified views of boxed regions. White dotted lines outline the cervical loop. Yellow arrows indicate positive signals. Scale bars, 50 μm. **e** Western blot and quantification of p-CaMKII and CaMKII in the proximal incisor region (mean ± SEM, *n* = 3; *P* = 0.014 0). **f** Heatmap showing expression of calcium signaling-related genes in control and *Gli1-CreER*^*T2*^*;Kdm6b*^*fl/fl*^ incisors (*n* = 3; individual *P*-values in Fig. S4c). **g**, **h** *Piezo1* in situ hybridization in control and *Gli1-CreER*^*T2*^*;Kdm6b*^*fl/fl*^ incisors. **g’**, **h’** Magnified views of boxed regions. White dotted lines outline the cervical loop. Yellow arrows indicate positive signals. Scale bars, 50 μm. **i** Schematic of time-lapse calcium imaging (Fluo-4) in dental mesenchymal cells. **j**, **k** Representative calcium traces from mesenchymal cells of control and *Gli1-CreER*^*T2*^*;Kdm6b*^*fl/fl*^ mice before and after Yoda1 stimulation. Each trace represents one cell (*n* = 15). **l** Quantification of spontaneous calcium spikes calculated as [(F_max_-F_0_)/F_0_] (mean ± SEM, *n* = 15; *P* = 0.006 9)

To investigate the downstream mechanisms by which *Kdm6b* deletion disrupts TAC fate and tissue homeostasis in response to mechanical stress, we performed bulk RNAseq on the proximal region of incisors from *Kdm6b*^*fl/fl*^ and *Gli1-CreER*^*T2*^*;Kdm6b*^*fl/fl*^ mice at 5 dpt. Hierarchical clustering analyses revealed clear separation between the gene expression profiles of control and *Gli1-CreER*^*T2*^*;Kdm6b*^*fl/fl*^ mice, with 1 603 differentially expressed genes identified (929 downregulated and 674 upregulated, FDR < 0.05; |fold change | ≥ 1.2) (Fig. 3a and Fig. S4a), consistent with the established role of KDM6B as a transcriptional activator. To further analyze the biological pathways affected, we performed Kyoto Encyclopedia of Genes and Genomes pathway (KEGG) enrichment analysis. The top 10 upregulated and downregulated pathways revealed enrichment in *Trp53* signaling, apoptosis, and PI3K-AKT signaling, consistent with the observed TAC apoptosis and proliferation defects. Notably, the calcium signaling pathway, which primarily lies downstream of mechanotransduction, was the most significantly upregulated (Fig. 3b and S4b). Given that intracellular calcium overload is a well-established trigger of apoptosis,^32^ we hypothesized that loss of *Kdm6b* may enhance cellular sensitivity to mechanical forces, thereby adversely affecting TAC fate and tissue homeostasis.

To explore this hypothesis in vivo, we first assessed levels of phosphorylated and total Calcium/Calmodulin-Dependent Protein Kinase II (CaMKII), a key functional readout of calcium signaling,^33^ via western blot and immunostaining. P-CaMKII was elevated in the TAC region of *Gli1-CreER*^*T2*^*;Kdm6b*^*fl/fl*^ mice (Fig. 3c–e), indicating heightened calcium responsiveness. Given the central role of calcium signaling in mechanotransduction, we next sought to identify the specific ion channels responsible for increased calcium influx in *Gli1-CreER*^*T2*^*;Kdm6b*^*fl/fl*^ mice. We quantified the mRNA levels of multiple calcium ion channels by quantitative real-time PCR (qRT-PCR), including mechanosensitive (PIEZO1, PIEZO2),^15^ voltage-gated (L, N, P, R, T types),^34^ and ligand-gated (TRPV family) types.^35^ Among these, *Piezo1* mRNA expression was significantly upregulated in *Gli1-CreER*^*T2*^*;Kdm6b*^*fl/fl*^ mice (Fig. 3f and Fig. S4c). In situ hybridization further confirmed increased *Piezo1* expression in the TAC region of *Gli1-CreER*^*T2*^*;Kdm6b*^*fl/fl*^ mice (Fig. 3g, h), suggesting that PIEZO1 contributes to calcium hyperactivity in *Gli1-CreER*^*T2*^*;Kdm6b*^*fl/fl*^ mice under high mechanical loading. To determine whether elevated *Piezo1* expression directly contributes to calcium hyperactivity in the *Gli1-CreER*^*T2*^*;Kdm6b*^*fl/fl*^ mice, we performed live-cell calcium imaging using the Fluo-4 calcium indicator dye in mesenchymal cells isolated from control and *Gli1-CreER*^*T2*^*;Kdm6b*^*fl/fl*^ mouse incisors. Cells were initially maintained in HBSS medium for 10 minutes to establish baseline calcium ion levels, followed by stimulation with the PIEZO1 activator Yoda1 to assess PIEZO1-specific calcium ion influx. Live-cell time-series imaging revealed a rapid and pronounced increase in calcium ion influx in the *Gli1-CreERT2;Kdm6b*^*fl/fl*^ group, with significantly higher maximum fluorescence intensity than controls (Fig. 3i–l). These findings confirmed that PIEZO1 overexpression leads to dysregulated calcium signaling in *Gli1-CreER*^*T2*^*;Kdm6b*^*fl/fl*^ mice. Taken together, these results highlight a critical role for KDM6B in calibrating PIEZO1 activity, serving as an essential epigenetic safeguard of mechanotransduction in load-bearing tissues.

### KDM6B regulates TAC fate and tissue homeostasis via H3K27me3-mediated control of *Piezo1* expression

**Fig. 4:**
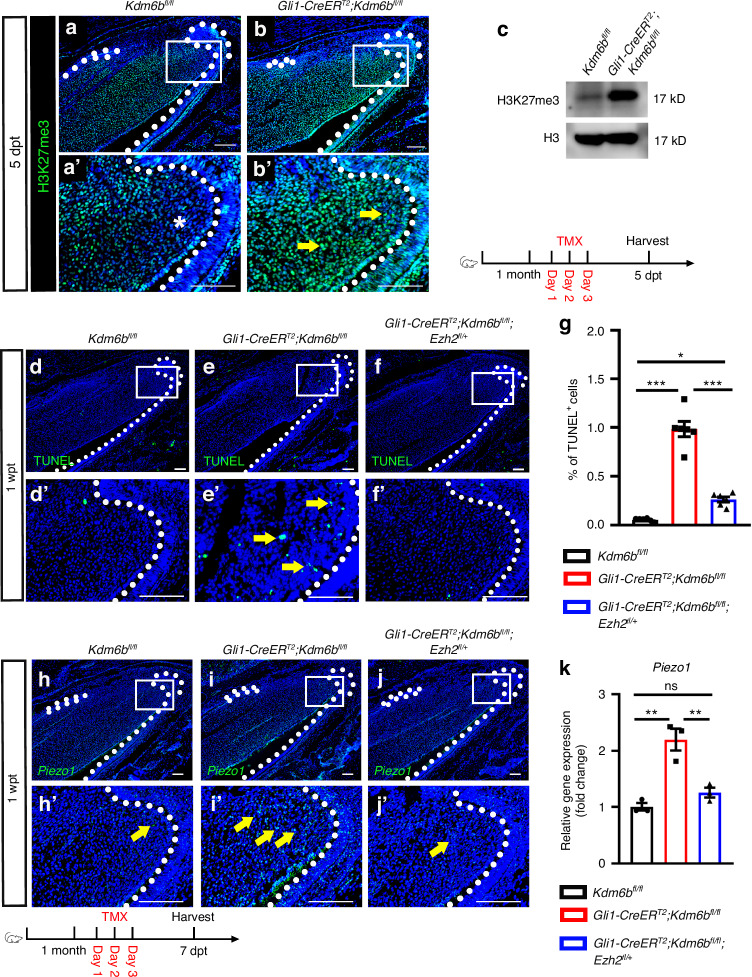
KDM6B regulates PIEZO1 expression and tissue homeostasis through H3K27me3. **a**, **b** H3K27me3 immunofluorescence in control and *Gli1-CreER*^*T2*^*;Kdm6b*^*fl/fl*^ incisors. **a’**, **b’** magnified views of boxed regions. White dotted lines outline the cervical loop. Asterisk indicates absence of signal. Yellow arrows indicate positive cells. Scale bars, 50 μm. **c** Western blot analysis of H3K27me3 and H3 in the proximal incisor region. **d–f** TUNEL staining in control, *Kdm6b* mutant, and *Ezh2* rescue incisors at 1 wpt. **d’–f’** Magnified views of boxed regions. White dotted lines outline the cervical loop. Yellow arrows indicate TUNEL^+^ cells. Scale bars, 50 μm. **g** Quantification of TUNEL^+^ cells in the dental mesenchymal region (mean ± SEM, *n* = 6; control vs. *Kdm6b* mutant, *P* < 0.000 1; *Kdm6b* mutant vs. *Ezh2* rescue, *P* < 0.000 1; control vs. *Ezh2* rescue, *P* = 0.019 7). **h–j** *Piezo1* in situ hybridization in the incisors of control, *Kdm6b* mutant, and *Ezh2* rescue mice. **h’–j’** Magnified views of boxed regions. White dotted lines outline the cervical loop. Yellow arrows indicate positive signals. **k** Relative *Piezo1* mRNA expression (mean ± SEM. *n* = 3; control vs *Kdm6b* mutant, *P* = 0.001 5; *Kdm6b* mutant vs *Ezh2* rescue, *P* = 0.004 9; control vs *Ezh2* rescue, *P* = 0.405 4. *Kdm6b* mutant: *Gli1-CreER*^*T2*^*;Kdm6b*^*fl/fl*^ ; *Ezh2* rescue: *Gli1-CreER*^*T2*^*;Kdm6b*^*fl/fl*^*;Ezh2*^*fl/+*^)

KDM6B has dual functional regulatory roles as a H3K27me3 demethylase and as a transcription activator controlling downstream gene expression.^23^ To determine the mechanism by which *Kdm6b* regulates *Piezo1* expression, we first compared H3K27me3 distribution and histone modification levels in the incisor of control and *Gli1-CreER*^*T2*^*;Kdm6b*^*fl/fl*^ mice using immunostaining and western blot. In control incisors, H3K27me3 was mainly found in the MSC region, epithelium, and dental pulp. In contrast, *Gli1-CreER*^*T2*^*;Kdm6b*^*fl/fl*^ mice showed a notable rise in H3K27me3 levels, particularly in the TAC region (Figs. 4a–c and S4d), indicating that KDM6B may regulate TAC fate determination through its H3K27me3 demethylase function.

Given that EZH1/2 and KDM6B function antagonistically to regulate H3K27me3 levels,^17^ we sought to test the hypothesis that loss of *Kdm6b* led to an increase in *Ezh2* and aberrantly increased H3K27me3 levels in the *Gli1-CreER*^*T2*^*;Kdm6b*^*fl/fl*^ mice. ScRNA-seq data revealed that *Ezh2*, not *Ezh1*, is preferentially enriched in the TAC region of the mouse incisor (Fig. S4e). Therefore, we generated *Gli1-CreER*^*T2*^*;Kdm6b*^*fl/fl*^*;Ezh2*^*fl/+*^ mice, in which *Ezh2* haploinsufficiency reduces the H3K27me3 level in *Gli1-CreER*^*T2*^*;Kdm6b*^*fl/fl*^ mice. We first confirmed that H3K27me3 levels in the TAC region were restored in *Gli1-CreER*^*T2*^*;Kdm6b*^*fl/fl*^*;Ezh2*^*fl/+*^ mice (Fig. S5a–c). MicroCT imaging and H&E staining further demonstrated that pathological expansion of the dental pulp cavity and thinner dentin observed in *Gli1-CreER*^*T2*^*;Kdm6b*^*fl/fl*^ mice were reversed in *Gli1-CreER*^*T2*^*;Kdm6b*^*fl/fl*^*;Ezh2*^*fl/+*^ mice (Fig. S5d–j), indicating restoration of normal tissue architecture.

To determine whether TAC apoptosis was also rescued, we performed TUNEL staining in control, *Gli1-CreER*^*T2*^*;Kdm6b*^*fl/fl*^, and *Gli1-CreER*^*T2*^*;Kdm6b*^*fl/fl*^*;Ezh2*^*fl/+*^ mice. The results showed that apoptosis levels in the TAC region were partially restored (Fig. 4d–g), further supporting the role of H3K27me3 regulation in TAC fate maintenance and determination. Next, we investigated whether KDM6B modulates *Piezo1* expression through H3K27me3 regulation. In situ hybridization and qRT-PCR analysis revealed that *Piezo1* expression, which was significantly upregulated in the TAC region of *Gli1-CreER*^*T2*^*;Kdm6b*^*fl/fl*^ mice, was restored to control levels in *Gli1-CreER*^*T2*^*;Kdm6b*^*fl/fl*^*;Ezh2*^*fl/+*^ mice (Fig. 4h–k). Collectively, these findings demonstrate that manipulating the H3K27me3 level in *Gli1-CreER*^*T2*^*;Kdm6b*^*fl/fl*^ mice can restore the *Piezo1* expression level and rescue the TAC defects that compromise incisor tissue homeostasis, indicating that H3K27me3 remodeling is essential for mechanoadaptive progenitor fate decisions and tissue homeostasis.

### BMI1 mediates KDM6B/H3K27me3-dependent repression of *Piezo1*

**Fig. 5:**
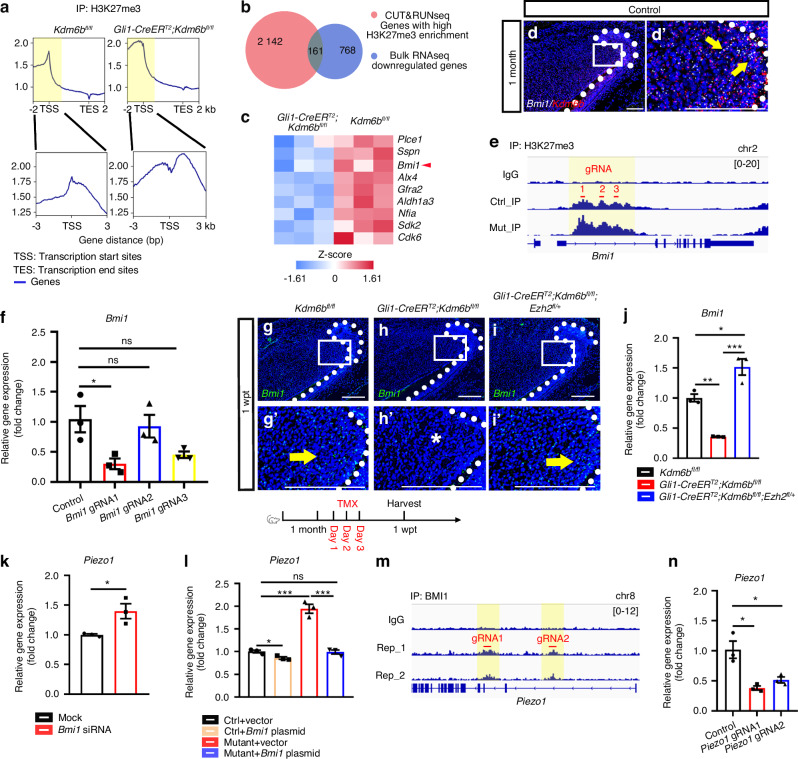
KDM6B epigenetically regulates BMI1 to silence PIEZO1 expression and maintain tissue homeostasis. **a** CUT&RUN profiles of H3K27me3 in the proximal incisor region of control and *Gli1-CreER*^*T2*^*;Kdm6b*^*fl/fl*^ mice at 5 dpt. TSS: Transcription start sites. TES: Transcription end sites. **b** Venn diagram showing overlap between genes with increased H3K27me3 enrichment in CUT&RUN and downregulated genes in RNA-seq from *Gli1-CreER*^*T2*^*;Kdm6b*^*fl/fl*^ incisors. **c** Heatmap of overlapping genes expressed in TACs in control and *Gli1-CreER*^*T2*^*;Kdm6b*^*fl/fl*^ mice. **d** Co-staining of *Bmi1* (white) and *Kdm6b* (red) in the wild-type mouse incisors. **d’** Magnified view of boxed region. White dotted lines outline the cervical loop. Yellow arrows indicate double-positive cells. Scale bars, 50 μm. **e** CUT&RUN tracks showing H3K27me3 enrichment at the *Bmi1* locus in control and *Gli1-CreER*^*T2*^*;Kdm6b*^*fl/fl*^ incisors. IgG serves as a negative control. Red lines indicate CRISPRi gRNA target sites. **f** RT-qPCR analysis of *Bmi1* expression following CRISPRi targeting (mean ± SEM, *n* = 3; gRNA1, *P* = 0.034 6; gRNA2, *P* = 0.709 1; gRNA3, *P* = 0.058 7). **g–i** *Bmi1* in situ hybridization in control, *Kdm6b* mutant, and *Ezh2* rescue incisors. **g’–i’** Magnified views of boxed regions. White dotted lines outline the cervical loop. Yellow arrows indicate positive signals. Asterisk indicates absence of signal. **j** Relative *Bmi1* mRNA expression in the three genotypes (mean ± SEM, *n* = 3; control vs *Kdm6b* mutant, *P* = 0.004 2; *Kdm6b* mutant vs *Ezh2* rescue, *P* = 0.000 2; control vs *Ezh2* rescue, *P* = 0.012 8). **k** *Piezo1* expression in dental mesenchymal cells after 3 days of control or *Bmi1* siRNA treatment (mean ± SEM, *n* = 3; *P* = 0.036 4). **l** *Piezo1* expression after vector or *Bmi1* plasmid treatment (mean ± SEM, *n* = 3; ctrl+vector vs. ctrl+*Bmi1*, *P* = 0.022 6; ctrl+vector vs. mutant+vector, *P* = 0.000 8; ctrl+vector vs. mutant+*Bmi1*, *P* = 0.838 4; mutant+vector vs. mutant+*Bmi1*, *P* = 0.000 9). **m** CUT&RUN profiles showing BMI1 enrichment at the *Piezo1* locus (IgG negative control and two BMI1 replicates). Red lines indicate CRISPRi gRNA target sites. **n** RT-qPCR analysis of *Piezo1* expression following CRISPRi targeting of BMI1-binding regions (mean ± SEM, *n* = 3; gRNA1, *P* = 0.012 3 ; gRNA2, *P* = 0.029 7). *Kdm6b* mutant: *Gli1-CreER*^*T2*^*;Kdm6b*^*fl/fl*^. *Ezh2* rescue: *Gli1-CreER*^*T2*^*;Kdm6b*^*fl/fl*^*;Ezh2*^*fl/+*^

*Kdm6b* promotes gene expression by demethylating H3K27me3. However, we observed increased *Piezo1* expression in *Gli1-CreER*^*T2*^*;Kdm6b*^*fl/fl*^ mice, suggesting that KDM6B does not directly suppress *Piezo1*, but rather modulates its expression indirectly via H3K27me3-mediated control of upstream transcriptional repressors. To identify potential intermediaries, we performed H3K27me3 CUT&RUN sequencing on proximal region of incisors from control and *Gli1-CreER*^*T2*^*;Kdm6b*^*fl/fl*^ mice. Using plotProfiles, we quantified the genome-wide distribution of H3K27me3 across gene bodies, from transcription start sites (TSS) to transcription end sites. In both groups, H3K27me3 was predominantly enriched near TSS, consistent with its role in promoter-associated repression. Notably, global H3K27me3 levels were elevated in the *Gli1-CreER*^*T2*^*;Kdm6b*^*fl/fl*^ mice, and a clear shift from a unimodal peak at the TSS (in controls) to a bimodal distribution flanking the TSS (in *Gli1-CreER*^*T2*^*;Kdm6b*^*fl/fl*^ mice) was observed (Fig. 5a). This pattern suggests altered chromatin architecture, with increased H3K27me3 deposition both upstream and downstream of the TSS in *Gli1-CreER*^*T2*^*;Kdm6b*^*fl/fl*^ mice, potentially leading to widespread transcriptional repression. We next identified 2 304 genes with significantly increased H3K27me3 occupancy in *Gli1-CreER*^*T2*^*;Kdm6b*^*fl/fl*^ mice, representing potential direct targets of KDM6B-mediated demethylation. Cross-referencing these with the 929 downregulated genes identified from bulk RNA-seq of *Gli1-CreER*^*T2*^*;Kdm6b*^*fl/fl*^ mouse incisors yielded 161 candidate genes likely repressed via increased H3K27me3 (Fig. 5b). To narrow down the candidates further, we analyzed their expression patterns using scRNA-seq data and identified nine genes expressed within the TAC region (Fig. 5c). Among these, BMI1 (B-lymphoma Mo-MLV insertion region 1) emerged as a prominent candidate due to its well-established role as a transcriptional repressor.^36^ Importantly, *Bmi1*^−/−^ mice exhibit phenotypes highly similar to those observed in *Gli1-CreER*^*T2*^*;Kdm6b*^*fl/fl*^ mice, including pathological dental pulp expansion and defects in dentin and enamel formation,^37,38^ further supporting our hypothesis that BMI1 may be a key intermediary linking KDM6B activity to *Piezo1* regulation.

To test our hypothesis, we first performed in situ hybridization analysis. Our result showed that *Bmi1* is expressed in both the epithelium and TAC region, where it colocalizes with *Kdm6b* (Fig. 5d). Consistently, CUT&RUN analysis demonstrated a marked increase in H3K27me3 occupancy at the *Bmi1* promoter in *Gli1-CreER*^*T2*^*;Kdm6b*^*fl/fl*^ mice, pinpointing a targeted region at Chr2: 18 677 783–18 680 811 (Fig. 5e). To determine whether H3K27me3 directly suppresses *Bmi1* transcription through this region, we performed CRISPR interference (CRISPRi) targeting the identified locus. Based on the CUT&RUN data, three gRNAs with high on-target efficiency and minimal predicted off-target effects were designed (Fig. 5e). CRISPRi-mediated repression resulted in a significant reduction of *Bmi1* expression in the *Bmi1* gRNA1 group compared to the control vector (Fig. 5f), indicating that aberrant accumulation of H3K27me3 suppresses *Bmi1* transcription via region 1. Consistent with this epigenetic regulation, *Bmi1* expression was markedly reduced in incisors from *Gli1-CreER*^*T2*^*;Kdm6b*^*fl/fl*^ mice and restored in *Gli1-CreER*^*T2*^*;Kdm6b*^*fl/fl*^*;Ezh2*^*fl/+*^ rescue mice, as confirmed by in situ hybridization and qRT-PCR (Fig. 5g–j). Together, these data establish *Bmi1* as a direct epigenetic target of H3K27me3 downstream of KDM6B.

To test the functional significance of BMI1 in incisor mesenchymal cell fate, we cultured primary mesenchymal cells from the proximal incisor regions of control and *Gli1-CreER*^*T2*^*;Kdm6b*^*fl/fl*^ mice. We transfected control mesenchymal cells with three types of *Bmi1*-targeting siRNA to knock down *Bmi1* expression and selected the most efficient siRNA for further experiments (Fig. S6a). The TUNEL staining showed a marked increase in apoptosis (TUNEL^+^ cells) (Fig. S6b–d), indicating that downregulated *Bmi1* contributes to apoptosis. Conversely, using *Bmi1* plasmid, *Bmi1* overexpression in mesenchymal cells from *Gli1-CreER*^*T2*^*;Kdm6b*^*fl/fl*^ mice rescued the increase in apoptosis seen in *Gli1-CreER*^*T2*^*;Kdm6b*^*fl/fl*^ mice, with a significant reduction in TUNEL^+^ cells compared to the mutant + vector group (Fig. S6e–j). To determine whether BMI1 regulates *Piezo1* expression, we performed real-time PCR following *Bmi1* knockdown and overexpression in vitro. The results revealed that *Piezo1* expression increased in *Bmi1*-knockdown mesenchymal cells (*Bmi1* siRNA) and decreased in *Bmi1*-overexpressing mesenchymal cells (Fig. 5k, l), suggesting transcriptional repression. To elucidate whether this regulation is direct, we performed CUT&RUN sequencing of BMI1 using the proximal incisor region of control mice. PlotProfiles revealed consistent peaks across replicates, with strong enrichment at promoter regions (Fig. S6k). The analysis revealed two strong BMI1-binding peaks in the intronic region of *Piezo1*, spanning the second and third exons (Fig. 5m), suggesting direct binding and transcriptional repression. To functionally validate these binding sites, we employed CRISPRi targeting each region using two gRNAs designed for high specificity (Fig. 5m). CRISPRi-mediated targeting resulted in a significant reduction of *Piezo1* expression in both gRNA1 and gRNA2 groups compared to controls (Fig. 5n), demonstrating that BMI1 directly suppresses *Piezo1* transcription through both intronic binding sites. Collectively, these findings reveal a KDM6B-H3K27me3-BMI1-PIEZO1 regulatory axis in which epigenetic repression of BMI1 enhances *Piezo1* expression, thereby linking chromatin regulation to mechanotransduction and incisor tissue homeostasis.

### Haploinsufficiency of *Piezo1* rescues TAC fate and tissue homeostasis in *Gli1-CreER*^*T2*^*;Kdm6b*^*fl/fl*^ mice

**Fig. 6:**
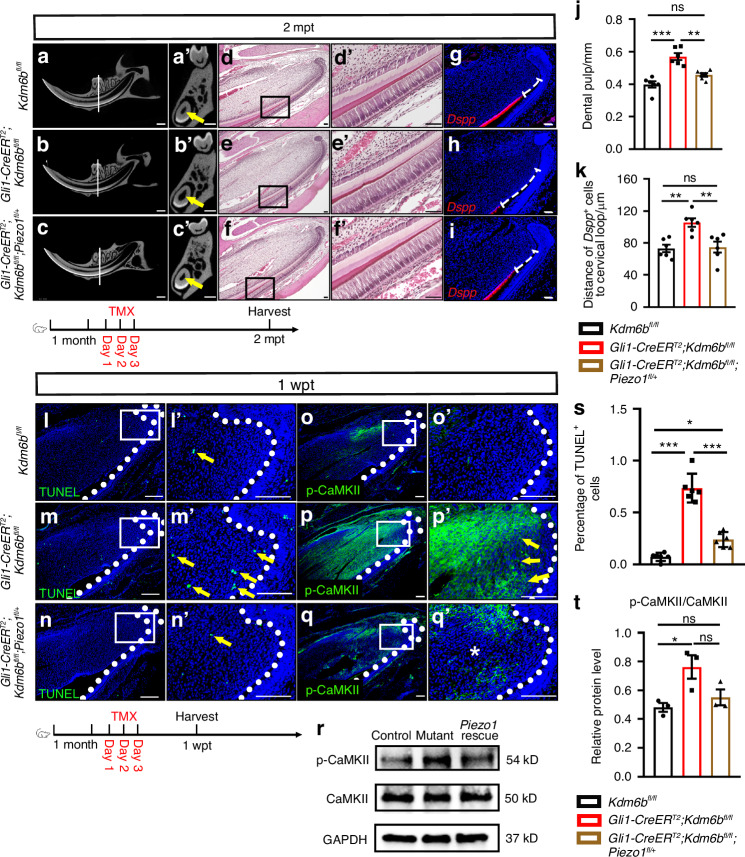
Haploinsufficiency of *Piezo1* rescues TAC fate and tissue homeostasis in *Gli1-CreER*^*T2*^*;Kdm6b*^*fl/fl*^ mice. **a–c** Micro-CT images of incisors from control, *Kdm6b* mutant, and *Piezo1* rescue mice at 2 mpt. **a’–c’** Coronal sections through the first molar furcation (white lines). Yellow arrows indicate dental pulp. Scale bars, 1 mm. **d–f** H&E staining of incisors from the three groups. **d’–f’** Magnified views of boxed regions. Scale bars, 50 μm. **g–i** *Dspp* (red) in situ hybridization (red) in the incisors from the three groups. White dotted lines show the distance between the cervical loop bending point and the odontoblast initiation. Scale bars: 50 µm. **j** Quantification of pulp cavity diameter (mean ± SEM, *n* = 6; control vs. *Kdm6b* mutant, *P* < 0.000 1; *Kdm6b* mutant vs. *Piezo1* rescue, *P* = 0.001 1; control vs. *Piezo1* rescue, *P* = 0.078 6). **k** Quantification of the distance between *Dspp*^+^ cells and the cervical loop (mean ± SEM. *n* = 6; control vs. *Kdm6b* mutant, *P* = 0.003 0; *Kdm6b* mutant vs. *Piezo1* rescue, *P* = 0.004 4; control vs. *Piezo1* rescue, *P* = 0.977 8). **l**–**n** TUNEL staining in the incisors from the three groups at 1 wpt. **l’–n’** Magnified views of boxed regions. White dotted lines outline the cervical loop. Yellow arrows indicate TUNEL^+^ cells. Scale bars: 50 µm. **o–q** Immunostaining of p-CaMKⅡ in the incisors of the three groups at 1 wpt. **o’–q’** Magnified views of boxed regions. White dotted lines outline the cervical loop. Yellow arrows indicate the positive signals. Scale bars: 50 µm. **r** Western blot of p-CaMKⅡ, CaMKⅡ, and GAPDH in the proximal regions of the incisors from the three groups. **s** Quantification of TUNEL^+^ cells in dental mesenchymal region (mean ± SEM. *n* = 6; control vs. *Kdm6b* mutant, *P* < 0.000 1; *Kdm6b* mutant vs. *Piezo1* rescue, *P* < 0.000 1; control vs. *Piezo1* rescue, *P* = 0.019 5). **t** Quantification of p-CaMKⅡ and CaMKⅡ protein levels (mean ± SEM. *n* = 3; control vs. *Kdm6b* mutant, *P* = 0.036 0; *Kdm6b* mutant vs. *Piezo1* rescue, *P* = 0.101 2; control vs. *Piezo1* rescue, *P* = 0.699 0). *Kdm6b* mutant: *Gli1-CreER*^*T2*^*;Kdm6b*^*fl/fl*^. *Piezo1* rescue: *Gli1-CreER*^*T2*^*;Kdm6b*^*fl/fl*^*;Piezo1*^*fl/+*^

To investigate the functional role of PIEZO1 as a downstream effector of KDM6B, and whether *Piezo1* upregulation drives the mechanoadaptive defects observed in *Gli1-CreER*^*T2*^*;Kdm6b*^*fl/fl*^ mice, we generated *Gli1-CreER*^*T2*^*;Kdm6b*^*fl/fl*^*;Piezo1*^*fl/+*^ mice as a rescue model with reduced *Piezo1* expression. Following tamoxifen induction at 1 month of age, we confirmed the *Piezo1* level was restored in the *Gli1-CreER*^*T2*^*;Kdm6b*^*fl/fl*^*;Piezo1*^*fl/+*^ mice (Fig. S6l). We observed that the pathologically expanded dental pulp cavity in *Gli1-CreER*^*T2*^*;Kdm6b*^*fl/fl*^ mice was restored to near wild-type dimensions in *Gli1-CreER*^*T2*^*;Kdm6b*^*fl/fl*^*;Piezo1*^*fl/+*^ mice, as evidenced by microCT imaging (Fig. 6a–c, j). Histological analysis further confirmed the rescue of dentin defects in the *Gli1-CreER*^*T2*^*;Kdm6b*^*fl/fl*^*;Piezo1*^*fl/+*^ mouse incisors (Fig. 6d–f). To assess the functional recovery of GLI1-derived TACs, we evaluated apoptosis and differentiation using TUNEL and *Dspp* staining. In the rescue mice, both the elevated apoptosis and delayed differentiation observed in *Gli1-CreER*^*T2*^*;Kdm6b*^*fl/fl*^ mice were significantly alleviated (Fig. 6g–i, k–n, s), indicating that reduced *Piezo1* expression is sufficient to restore TAC homeostasis. To further check the calcium ion activity in the *Piezo1* rescue model, we detected the protein levels of p-CaMKII by immunostaining and western blot in control, *Gli1-CreER*^*T2*^*;Kdm6b*^*fl/fl*^, and *Gli1-CreER*^*T2*^*;Kdm6b*^*fl/fl*^*;Piezo1*^*fl/+*^ mice at 1 wpt. The results showed that the calcium hyperactivity in *Gli1-CreER*^*T2*^*;Kdm6b*^*fl/fl*^mice was also restored to a normal level in *Gli1-CreER*^*T2*^*;Kdm6b*^*fl/fl*^*;Piezo1*^*fl/+*^ mouse incisors (Fig. 6o–r, t). Taken together, these results establish that PIEZO1 hyperactivation is a key mediator of the mechanoadaptive defects observed in *Kdm6b*-deficient mice. Genetic attenuation of *Piezo1* rescues TAC survival, differentiation, and tissue homeostasis, highlighting a KDM6B-BMI1-PIEZO1 regulatory axis that safeguards progenitor fate under mechanical stress. This work reveals a critical epigenetic mechanism for maintaining tissue homeostasis in load-bearing environments, with potential therapeutic implications for mechanosensitive tissues.

## Discussion

**Fig. 7:**
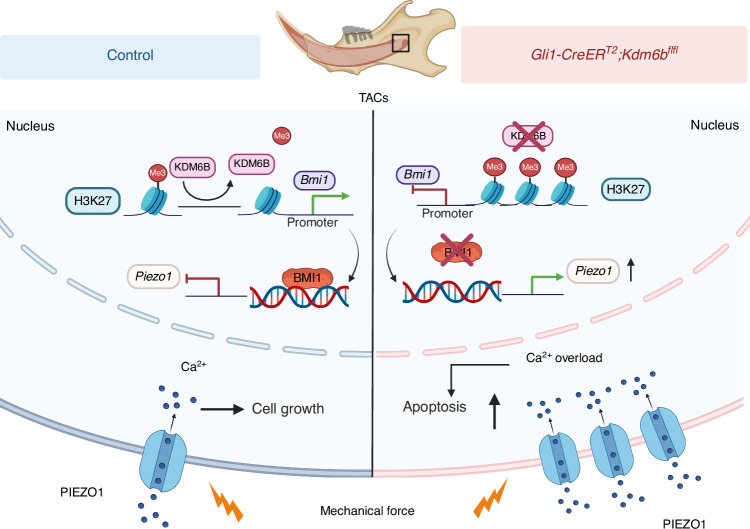
Schematic representation of KDM6B safeguarding tissue homeostasis to mechanical stress through epigenetic control of PIEZO1-mediated mechanotransduction. Using the mouse incisor as a model of mechanical loading, we reveal that within TACs, *Kdm6b* demethylates H3K27me3, thereby relieving the repression of the *Bmi1* gene. Normal BMI1 inhibits *Piezo1* expression. This maintains physiological PIEZO1 levels, ensuring calibrated Ca^2+^ influx for proliferation and differentiation. In contrast, loss of *Kdm6b* leads to an accumulation of H3K27me3 at the *Bmi1* promoter region, which silences *Bmi1* expression and diminishes BMI1 formation. This reduction results in pathologically increased PIEZO1 ion channels in the membrane. The subsequent Ca^2+^ overload triggers TAC apoptosis while reducing proliferation and differentiation. Ultimately, these molecular events compromise tissue homeostasis. *Schematic created with BioRender.com. Ho, T. (2026)* https://BioRender.com/8mzv4a3

Our study reveals an essential role for the epigenetic regulator KDM6B in controlling mineralized tissue homeostasis. Using in vivo mouse incisor models with varying degrees of loading, we demonstrate that KDM6B safeguards force-induced TAC damage and tissue homeostasis by modulating PIEZO1-dependent mechanotransduction. Mechanistically, KDM6B demethylates H3K27me3 at the *Bmi1* promoter, sustaining BMI1 expression, which in turn represses *Piezo1*. This cascade limits PIEZO1 channel abundance at the plasma membrane, restricting calcium influx to levels that support TAC proliferation and differentiation. In contrast, loss of *Kdm6b* leads to excessive H3K27me3 accumulation and transcriptional silencing of *Bmi1*, resulting in de-repression of *Piezo1*, calcium overload, TAC apoptosis, and compromised incisor tissue homeostasis (Fig. 7). Notably, haploinsufficiency of *Piezo1* in *Gli1-CreER*^*T2*^*;Kdm6b*^*fl/fl*^*;Piezo1*^*fl/+*^ mice rescues calcium homeostasis, TAC survival, and overall tissue architecture and organization. Our findings thus establish a KDM6B-H3K27me3-BMI1-PIEZO1 axis critical for mechanoadaptive tissue homeostasis regulation.

Tissue regeneration relies on a dynamic stem cell microenvironment in which mechanical cues generated by factors such as extracellular matrix stiffness, tissue strain, and cellular contractility play a central role in modulating cell behavior.^12–14,32,39^ Despite increasing recognition of mechanotransduction’s roles in development and disease, how stem and progenitor cells maintain homeostasis under mechanical load remains poorly understood. Here, we show that KDM6B is required to calibrate mechanosensitive calcium signaling in TACs, integrating epigenetic regulation with ion channel activity to preserve the regenerative potential of mechanically active tissues. Our findings also align with previous studies showing that EZH2, a methyltransferase that deposits H3K27me3, is downregulated under mechanical stress,^22^ suggesting that the reduction of repressive chromatin marks could be a universal mechanism enabling cellular adaptation to force. Interestingly, H3K27me3 has also been implicated in stress adaptation in plants,^21^ pointing to a conserved role for chromatin-based buffering mechanisms across multiple biological systems. Thus, our data suggest that histone demethylation via KDM6B functions as a cellular “mechanostat,” tuning transcriptional programs in response to biomechanical stimuli. These findings open new avenues for therapeutic intervention, suggesting that modulation of chromatin states could help mitigate tissue damage and enhance regenerative capacity in mechanically stressed environments.

Tissue turnover and regeneration follow a hierarchical model in which stem cells give rise to TACs, which then differentiate into specialized cell types during stem cell renewal.^2,15,29^ However, how different cell populations within this hierarchy respond to mechanical force remains unclear. Here, we demonstrate that *Kdm6b* deletion specifically induces TACs apoptosis under mechanical loading, disrupting feedback to MSCs and impairing tissue growth. Previous studies have shown that TACs serve as a critical signaling hub essential for feeding back to MSCs, maintaining tissue homeostasis, and guiding overall tissue function.^40–42^ Compared to MSCs or differentiated cells, TACs exhibit rapid proliferation, which may render them particularly susceptible to mechanical stress. Our findings position TACs as a mechanosensitive node within the stem cell hierarchy, and suggest that their resilience is essential for sustaining long-term tissue renewal. Similar vulnerabilities have been described in other systems, such as the METTL3-dependent survival of intestinal TACs,^43^ emphasizing the importance of context-specific regulation in transient progenitor populations, which is likely to be of significance for the clinical translation of these findings.

Among the known networks of mechanotransduction, including focal adhesion, caveolae, and cytoskeletal tension,^12^ PIEZO1 has emerged as a key mechanosensory ion channel that directly converts mechanical forces into intracellular calcium signals. Unlike adhesion-based mechanotransducers, PIEZO1 responds rapidly to membrane tension and curvature, enabling cells to sense both dynamic and sustained mechanical cues. Accordingly, PIEZO1 is broadly involved in orchestrating a wide range of physiological processes, stem cell regulation, epithelial integrity, and tissue regeneration.^32,44–46^ In skeletal and dental tissues, recent studies have established PIEZO1 as a central mechanostat in osteoblasts, osteocytes, mesenchymal stem cells, and periodontal ligament stem cells, where it regulates osteogenic differentiation, bone mass maintenance, alveolar bone remodeling, and adaptive responses to orthodontic or occlusal forces through downstream activation of β-catenin/LARS2, YAP/TAZ, and YAP/β-catenin pathways.^47–49^ Consistent with its essential physiological role, aberrant PIEZO1 activity has been implicated in several human and murine pathologies, including cardiovascular disorders, osteoarthritis, and skeletal fragility.^10,50–52^ In the context of bone formation and fracture healing, genetic loss of *Piezo1* in osteogenic or periosteal progenitor populations results in impaired load-induced bone formation, defective endochondral ossification, and delayed repair, whereas pharmacological activation of PIEZO1 enhances osteogenesis and accelerates fracture healing.^47,48,53^ These findings highlight the importance of precisely tuned PIEZO1 activity for skeletal adaptation and regeneration. While mechanical forces typically experienced under physiological conditions modulate stem cell behavior through PIEZO1 activation, excessive force can saturate PIEZO1 channels, preventing further modulation.^15^ This observation suggests that stem and progenitor cells possess intrinsic protective mechanisms to buffer against mechanical overload, one of which may involve limiting PIEZO1 channel abundance at the plasma membrane. However, the upstream regulatory mechanisms controlling PIEZO1-dependent mechanotransduction remain poorly understood. Our study reveals that KDM6B regulates *Piezo1* transcription in TACs via H3K27me3-dependent repression of *Bmi1*. By maintaining BMI1 expression, KDM6B indirectly suppresses PIEZO1 levels, thereby restraining calcium influx and protecting TACs from apoptosis under mechanical stress. This epigenetic control of ion channel expression links chromatin remodeling to mechanotransduction, providing a mechanistic framework for how tissue progenitors adapt to physical forces. Importantly, our results align with emerging evidence that mechanotransduction is intimately coupled to chromatin regulation. Beyond PIEZO1, KDM6B may function as a broader epigenetic integrator of mechanical cues, modulating chromatin accessibility downstream of force-responsive pathways. Indeed, extracellular matrix stiffness regulates KDM6B expression through YAP/TAZ signaling and enhances its demethylase activity in mechanically stimulated contexts.^54^ Conversely, PIEZO1-mediated mechanosignaling can remodel the epigenetic landscape, as exemplified by suppression of H3K9me3 during goblet cell differentiation and mucus barrier formation.^55^ Together, these observations support a model of bidirectional crosstalk between mechanotransduction and chromatin dynamics, highlighting how cells integrate mechanical inputs into durable transcriptional programs and potentially encode mechanical history during tissue development, regeneration, and disease. The broader roles of epigenetic regulators in coordinating mechanotransduction remain largely unexplored and warrant further investigation.

Our work also highlights BMI1 as a key mediator of this axis. Although BMI1 is well known for repressing *p16* and maintaining self-renewal in various stem cell compartments,^56,57^ How BMI1 is regulated and what is its downstream effector in controlling the fate of stem cell, are not well understood. Here we have discovered that KDM6B is an upstream regulator of *Bmi1*, which represses *Piezo1* as a downstream target in maintaining mineralized tissue homeostasis. We show that BMI1 binds directly to the intronic regions of *Piezo1*, and that its silencing results in PIEZO1 overexpression and TAC apoptosis. While BMI1 is known as a cell cycle regulator, we establish that it is also a gatekeeper of mechanotransduction, essential for protecting stem cell-derived progenitors under stress.

In conclusion, our study uncovers a previously unrecognized role for KDM6B in mechanoadaptive mineralized tissue homeostasis. Using in vivo models, our findings position KDM6B as a molecular “mechanostat” that buffers mechanical inputs, suggesting a generalizable mechanism by which epigenetic regulators in mineralized tissues coordinate force-adaptive responses. This work expands current models of skeletal and dental mechanobiology by revealing how epigenetic control of ion channel expression safeguards tissue progenitors in mechanically dynamic environments and provides a conceptual basis for understanding how cells integrate, adapt to, and remember mechanical histories. Over the past decade, regenerative medicine has made remarkable strides, with increasing attention to how the physical microenvironment directs stem cell behavior and tissue repair.^18^ Beyond advancing our fundamental understanding of mechanotransduction, our findings suggest the KDM6B-BMI1-PIEZO1 axis as a therapeutic target that could be leveraged for protecting stem cell niches and enhancing regeneration in mechanically dynamic environments, such as the musculoskeletal and bone systems.

### Limitations

While our study identifies PIEZO1 hyperactivation as a major driver of TAC apoptosis in *Kdm6b*-deficient mice, some residual apoptosis persists despite *Piezo1* haploinsufficiency in *Kdm6b* deficient mice, suggesting that KDM6B may also regulate other stress pathways, such as the cell cycle.^23^ In addition, while mechanical stress was the focus of this study, how KDM6B-mediated chromatin remodeling responds to other environmental stimuli, such as temperature and hypoxia,^58^ remains to be explored. Future work will be necessary to further reveal how KDM6B may integrate different types of extrinsic cues into tissue-specific gene regulatory programs.

## Materials and Methods

### Animal studies

The following mouse lines were utilized in this study: *Gli1-CreER*^*T2*^ (JAX #007913), *Kdm6b*^*fl/fl* 17^, *Sox2-CreERT2* (JAX #017593), *Gli1-LacZ* (JAX #008211), *tdTomato* reporter mice (JAX #007905), *Ezh2*^*fl/fl*^(JAX #022616), and *Piezo1*^*fl/fl*^ (JAX #029213). To generate *Gli1-CreER*^*T2*^*;Kdm6b*^*fl/fl*^ mice, *Gli1-CreER*^*T2*^ mice were crossed with *Kdm6b*^*fl/fl*^ mice. Genomic DNA was isolated from ear tissue using DirectPCR solution (Viagen, 102 T), incubated at 55 °C overnight, and subsequently heat-inactivated at 85 °C for 60 min. PCR-based genotyping was performed using GoTaq Green Master Mix (Promega). All mice were housed under specific pathogen-free conditions and maintained on a mixed genetic background. All animal procedures were conducted in accordance with protocols approved by the University of Southern California Department of Animal Resources and Institutional Animal Care and Use Committee (IACUC).

### Tamoxifen administration

Tamoxifen (Sigma-Aldrich, T5648) was dissolved in corn oil (Sigma, C8267) at a concentration of 20 mg/mL. Mice were administered tamoxifen via intraperitoneal (i.p.) injection at a dose of 2 mg per 10 g of body weight daily for three consecutive days, beginning at one month of age.

### Sample collection

Mouse mandibles were dissected at various time points following tamoxifen administration and fixed overnight at 4 °C in 10% neutral buffered formalin (NBF; Sigma, HT501128). Samples were subsequently decalcified in 20% EDTA for 2–4 weeks. For paraffin embedding, samples were processed through a graded ethanol series and xylene, embedded in paraffin wax, and sectioned at 5 µm thickness. For cryosectioning, samples were dehydrated in 15% sucrose in DEPC-PBS, followed by 30% sucrose/50% OCT (Sakura, 4583), and then embedded in OCT compound on dry ice. Cryosections were cut at a thickness of 8 µm using a cryostat (Leica, CM3050S).

### Histological analysis, immunofluorescence staining, and in situ hybridization

For hematoxylin and eosin (H&E) staining, paraffin sections were processed according to standard protocols. For immunofluorescence analysis, sections were rinsed in PBS to remove OCT after incubation in a 60 °C oven for 3 h, followed by permeabilization with 1% Triton X-100 for 15 min. Sections were then blocked with blocking buffer (PerkinElmer, FP1012) for 1 h at room temperature and incubated overnight at 4 °C with primary antibodies. After washing with PBST, sections were incubated with Alexa Fluor 488-, 568-, or 680-conjugated secondary antibodies for 2 h at room temperature. Nuclei were counterstained with DAPI (Thermo Fisher Scientific, D1306) for 5 min, followed by mounting of the sections.

The primary and secondary antibodies used were: rabbit anti-Ki67 (1:100, Abcam, ab15580); chicken anti-β-galactosidase (1:100, Abcam, ab9361); rabbit anti-tri-methyl-histone H3 (Lys27) (C36B11) (1:100, Cell Signaling Technology, 9733); mouse anti-CaMKII (phospho T286) antibody [22B1] (1:50, Abcam, ab171095); Alexa Fluor 488 anti-rabbit (1:200, Thermo Fisher Scientific, A11008); Alexa Fluor 568 anti-rabbit (1:200, Thermo Fisher Scientific, A11036); and Alexa Fluor 488 anti-chicken (1:200, Thermo Fisher Scientific, A11039).

For RNA in situ hybridization, cryosections were air-dried at 60 °C for 4 h and processed using the RNAscope Multiplex Fluorescent Reagent Kit v2 (Advanced Cell Diagnostics, 323100) following the manufacturer’s instructions. RNAscope probes targeting *Kdm6a*, *Kdm6b*, *Uty*, *Dspp*, *Bmi1*, and *Piezo1* were used (Advanced Cell Diagnostics).

### Surgeries

Mechanical force loading models were established in 4-week-old *Kdm6b*^*fl/fl*^ and *Gli1-CreER*^*T2*^*;Kdm6b*^*fl/fl*^ mice, which had been treated with tamoxifen for seven days prior to the procedure. Following anesthesia with isoflurane, a notch was created on the labial surface of the mandibular incisor enamel near the gingival margin using a carbide bur (Brasseler USA, 018554U0). Incisor growth was monitored by measuring the movement of the notch over time.

For the mechanical hypo-loading model, *Kdm6b*^*fl/fl*^ and *Gli1-CreER*^*T2*^*;Kdm6b*^*fl/fl*^ mice were treated with tamoxifen for two days prior to surgery. The left mandibular incisor was clipped to approximately half its original length, and subsequent incisor growth was assessed based on the extent of regrowth from the clipping site. For the bilateral clipping model, both mandibular incisors were clipped to a similar extent at the same time to maintain symmetric loading conditions while inducing a transient injury. Postoperative photographs were taken daily until the lengths of the left and right mandibular incisors were of equal length and in contact with the maxillary incisors. All measurements were performed using ImageJ software.

### MicroCT analysis

Mouse mandibles were harvested as described above and subjected to micro-computed tomography (microCT). Scans were performed using a SCANCO mCT50 system (Scanco V1.28) at the University of Southern California Molecular Imaging Center. Imaging was conducted at a resolution of 10 µm, with the X-ray source set to 90 kVp and 78 µA.

### TUNEL assays

TUNEL staining was performed on cyrosections using the Click-iT™ Plus TUNEL Assay for In Situ Apoptosis Detection Kit (Thermo Fisher Scientific, C10617), according to the manufacturer’s protocol. Briefly, sections were rinsed in PBS to remove residual OCT compound and permeabilized with proteinase K in PBS for 15 min at room temperature. Subsequently, sections were incubated with the TdT reaction mixture at 37 °C for 1 h in a humidified chamber. After washing, incorporated nucleotides were detected using the Click-iT™ Plus detection cocktail. Nuclei were counterstained with DAPI, and sections were mounted with an antifade mounting medium.

### EdU incorporation and staining

For transit-amplifying cell (TAC) differentiation analysis, EdU (25 mg/kg body weight) was administered to *Kdm6b*^*fl/fl*^ and *Gli1-CreER*^*T2*^*;Kdm6b*^*fl/fl*^ mice via a single intraperitoneal (IP) injection at 12 days post-induction. Samples were collected 48 h following EdU injection. For the label-retaining cell assay, EdU was administered by daily IP injection beginning at PN5 and continuing for 4 weeks, followed by an 8-week chase period without EdU. Mandibles were then harvested, fixed, decalcified, and cryosectioned. EdU incorporation was detected using the Click-iT™ Plus EdU Cell Proliferation Kit (Invitrogen, C10637), according to the manufacturer’s instructions.

### RNA extraction and Quantitative Real-time PCR

Mouse incisors were dissected, and total RNA was isolated from the cervical loop region of *Kdm6b*^*fl/fl*^, *Gli1-CreER*^*T2*^*;Kdm6b*^*fl/fl*^, *Gli1-CreER*^*T2*^*;Kdm6b*^*fl/fl*^*;Ezh2*^*fl/+*^ and *Gli1-CreER*^*T2*^*;Kdm6b*^*fl/fl*^*;Piezo1*^*fl/+*^ mice one week after tamoxifen induction using the RNeasy Plus Micro Kit (QIAGEN, 74004) according to the manufacturer’s instructions. For each biological replicate, incisors from three mice were pooled, and three independent biological replicates were collected per genotype. Complementary DNA (cDNA) was synthesized using the iScript™ cDNA Synthesis Kit (Bio-Rad, 1708890). qRT-PCR was performed using SsoAdvanced™ Universal SYBR® Green Supermix (Bio-Rad, 1725201) on a CFX96 Real-Time PCR Detection System (Bio-Rad). Relative gene expression levels were normalized to β-actin, and primer sequences are listed in Table S1.

### RNA sequencing and data analysis

Incisor samples were collected five days after induction of one-month-old *Kdm6b*^*fl/fl*^ and *Gli1-CreER*^*T2*^*;Kdm6b*^*fl/fl*^ mice. For each sample, incisors from three mice were pooled. The proximal regions of the incisors were isolated for RNA extraction using the RNeasy Micro Kit (QIAGEN), following the manufacturer’s protocol. RNA quality was assessed using an Agilent 2100 Bioanalyzer, and only samples with RNA integrity numbers greater than 9.0 were used for sequencing. cDNA library preparation and sequencing were performed by DNA Link, Inc. Paired-end sequencing (50 cycles) was conducted on the NovaSeq SP platform for three biological replicates per group. Raw reads were quality-trimmed, aligned to the mm10 mouse reference genome using STAR (version 2.6.1 d), and normalized using the Upper Quartile method. Differentially expressed genes were identified based on a false discovery rate threshold of < 0.05. KEGG pathway enrichment analysis was subsequently performed on the set of differentially expressed genes. The original data are available in the FaceBase (DOI: 10.25550/88-ZJCW).

### Cell culture

Dental mesenchymal cells were cultured as previously described.^31^ Briefly, the proximal mesenchyme of the incisor was dissected and cut into small pieces. These minced tissues were seeded onto culture plates and cultured in α-MEM medium supplemented with 10% fetal bovine serum (FBS) and 1% penicillin/streptomycin. Cells were maintained in a humidified incubator at 37 °C with 5% CO₂.

### Calcium influx measurement

Dental mesenchymal cells were used to detect calcium ion activity. Intracellular calcium ion levels were assessed using Fluo-4 AM (Thermo Fisher Scientific, F14217), a calcium indicator dye, according to the manufacturer’s instructions. Cells were washed three times with Dulbecco’s phosphate-buffered saline (DPBS) and then incubated with Fluo-4 (5 µmol/L) for 30 min at 37 °C. After incubation, cells were washed three times with DPBS to remove excess dye. Fluorescence signals were captured using time-series imaging on a fluorescence microscope (Keyence BZ-X710). The initial 10-minute period was recorded to establish baseline fluorescence intensity, after which 50 µmol/L Yoda1(MedChemExpress, HY-18723) was added to the cells to activate PIEZO1 channels. Fluorescence signals were subsequently collected for an additional 50 min following Yoda1 addition. Fluorescence intensity was quantified using ImageJ software, and data were analyzed to assess changes in intracellular calcium levels over time.

### Western blot

For western blots, the proximal regions of incisors of *Kdm6b*^*fl/fl*^ and *Gli1-CreER*^*T2*^*;Kdm6b*^*fl/fl*^ mice were collected and homogenized using an ultrasonic homogenizer in T-PER™ Tissue Protein Extraction Reagent (ThermoFisher Scientific, 78510) supplemented with Protease and Phosphatase Inhibitor Cocktail (ThermoFisher Scientific, 78441) on ice. The lysates were centrifuged at 12 000 × g for 30 min at 4 °C to remove cell debris. Protein concentrations were determined using Quick Start™ Bradford 1x Dye Reagent (Bio-Rad, 5000205) following the manufacturer’s protocol. Equal amounts of protein (100 µg) were boiled at 95 °C for 5 min, then separated by SDS-PAGE on a 4%–15% precast polyacrylamide gel (Bio-Rad, 456-1084) and transferred to a 0.22 µm PVDF membrane (Millipore, IPVH00010). The membrane was blocked with 5% non-fat dry milk (Bio-Rad, 170-6404) in Tris-buffered saline with 0.1% Tween-20 (TBST) for 1 h at room temperature. The membrane was then incubated overnight at 4 °C with a primary antibody. After washing with TBST, the membrane was incubated with an HRP-conjugated secondary antibody (1:5 000) for 2 h at room temperature. Protein bands were visualized using an iBright 1500 (ThermoFisher Scientific).

The primary antibodies used were: rabbit anti-Histone H3 Tri-methyl (Lys27) (1:1 000, Cell Signaling Technology, 9733); rabbit anti-Histone H3 (D1H2) (1:1 000, Cell Signaling Technology, 4499); mouse anti-CaMKII (phospho T286) antibody [22B1] (1:500, Abcam, ab171095); mouse anti-CaMKII antibody (6G9) (1:1 000, Cell Signaling Technology, 50049); rabbit anti-GAPDH antibody (14C10) (1:1 000, Cell Signaling Technology, 2118).

### CUT&RUN sequence and analysis

Tissue samples from the harvested proximal regions of mouse incisors were enzymatically digested with collagenase I (4 mg/mL) and dispase II (2 mg/mL) and incubated at 37 °C for 15 minutes with shaking at 700 r/min, followed by filtration to obtain a single-cell suspension. For Cut&Run, 5 million cells per reaction were permeabilized using the digitonin-based buffer included in the CUTANA™ CUT&RUN Kit (CUTANA, 14-1048) as per the manufacturer’s instructions. The cell-bead mixtures were then incubated overnight at 4 °C with either IgG (negative control) or primary antibodies: anti-Histone H3 Tri-methyl (Lys27) (1:50, Cell Signaling Technology, 9733S) and BMI1 Polyclonal antibody (5 µg, Proteintech, 10832-1-AP). After primary antibody binding, pA-MNase was added and incubated for 1 hour at 4 °C. The cutting reaction was activated by the addition of the activation buffer and incubation at 37 °C for 30 minutes, enabling the pA-MNase to cleave unprotected chromatin regions. The reaction was stopped with the stop buffer, and the enriched DNA was purified using the provided reagents. DNA libraries were prepared from the purified DNA using the CUTANA™ CUT&RUN Library Prep Kit (CUTANA, 14-1001), followed by paired-end sequencing (50 cycles) on the NextSeq 10B platform (Illumina). Sequencing data were analyzed using the Galaxy platform. Raw sequencing files (FASTQ format) were first subjected to quality control using FastQC. The reads were then trimmed for adapter sequences and low-quality bases using Trim Galore, and subsequently aligned to the reference genome mm10 with Bowtie2. Aligned reads were processed using SAMtools for sorting and removal of duplicates. Peak calling was performed using MACS2 to identify regions of enriched protein-DNA interactions, followed by visualization of peak distributions in IGV. Functional annotation of the peaks was carried out using ChIPseeker, and peak distributions across gene regions, like transcriptional start sites (TSS), were visualized using the ComputeMatrix and PlotHeatmap tools. The original data are available in the FaceBase (DOI: 10.25550/88-ZJCW).

### siRNA and plasmid transfection

Dental mesenchymal cells were seeded in appropriate culture plates. For siRNA transfection, dental mesenchymal cells were plated in 4-well slides for TUNEL staining or 96-well plates for qRT-PCR and transfected with 10 nmol/L *Bmi1* siRNA (ThermoFisher Scientific, s63005, s63006, s63007; negative control siRNA, 4390843) using Lipofectamine™ RNAiMAX Transfection Reagent (Thermo Fisher Scientific, 13778075). After 3 days, the cells were processed for TUNEL staining and qRT-PCR analysis. For plasmid transfection, cells were transfected with 1 µg/µL *Bmi1* (Myc-DDK-tagged) plasmid (OriGene, MR226936; vector, PS100001) DNA using Lipofectamine™ 3000 Transfection Reagent (Thermo Fisher Scientific, L3000150) for 3 days, followed by TUNEL staining and qPCR. The sequences of qPCR primers are provided in Table S1.

### CRISPRi vector design and transfection

To check the H3K27me3-*Bmi1* regulatory interaction, three guide RNA sequences were designed to target the H3K27me3-enriched regions identified at the *Bmi1* locus (Table S1). These gRNAs were cloned into the pCas-Guide-Puro-CRISPRi vector to generate a custom *Bmi1* CRISPRi kit (Origene Technologies, CW312379), which was constructed by Origene Technologies. To examine BMI1 binding to *Piezo1*, two gRNA sequences were designed to target the two BMI1-binding sites identified by CUT&RUN (Table S1). These gRNAs were similarly cloned into the pCas-Guide-Puro-CRISPRi vector to generate a custom *Piezo1* CRISPRi kit (Origene Technologies, CW312380). The pCas-Guide-Puro-CRISPRi-Scramble vector (Origene Technologies, GE100084) was used as a negative control.

Primary cells isolated from control mouse incisors were transfected with the indicated CRISPRi constructs using TurboFectin 8.0 Transfection Reagent (Origene Technologies, TF81001). Following transfection, total RNA was extracted, reverse-transcribed into cDNA, and subjected to qRT-PCR analysis to assess *Bmi1* and *Piezo1* expression levels.

### Single-cell RNA sequencing analysis

To analyze our lab previous scRNA-seq data from wild-type mouse incisors(GSE237305),^31^ we first downloaded gene-barcode matrix files: matrix.mtx, barcodes.tsv, and features.tsv, from the GEO database. These files were imported into the Seurat package (R) using the Read10X function to create a Seurat object for downstream analysis. We performed standard quality control by filtering out cells with low gene counts, high mitochondrial content, or suspected doublets. The dataset was normalized using NormalizeData, and highly variable genes were identified with FindVariableFeatures. We then scaled the data, conducted dimensionality reduction using PCA, and applied graph-based clustering (FindNeighbors and FindClusters), followed by UMAP for visualization. Cell populations were annotated based on previously reported marker genes.^31^ To examine the expression of key genes involved in epigenetic regulation, we used FeaturePlot to visualize expression across clusters and cell types.

### Image analysis

Images of all stained sections were captured using a Keyence BZ-X710/BZ-X810 system. Quantitative image analysis was performed using ImageJ.

### Quantification and statistical analysis

GraphPad Prism Software was used for all statistical analyses. Data are shown as individual points and mean ± SEM. *N* ≥ 3 for all samples unless otherwise stated. Unpaired *t*-tests with two-tailed calculations were used for the comparison of two groups, and one-way ANOVA was used for other comparisons. *P* < 0.05 was considered statistically significant.

## Supplementary information

supplementary
