# Supplementary material for: KDM6B safeguards mineralized tissue homeostasis from mechanical stress through epigenetic control of PIEZO1-mediated mechanotransduction in the mouse incisor
Source: Bone Res. 2026 May 28;14:59. doi: 10.1038/s41413-026-00544-2 (PMC13219498; doi:10.1038/s41413-026-00544-2)
Supplement: Supplementary file 1 — supplementary [file 41413_2026_544_MOESM1_ESM.docx]

Supplementary Materials for

**KDM6B safeguards mineralized tissue homeostasis from mechanical stress through epigenetic control of PIEZO1-mediated mechanotransduction in the mouse incisor**

Lin Meng^1^, Mingyi Zhang^1^, Jifan Feng^1^, Tingwei Guo^1^, Hana Hekmat^1^, Heliya Ziaei^1^, Peng Chen^1^, Aaron Harouni^1^, Thach-Vu Ho^1^, and Yang Chai*1

1 Center for Craniofacial Molecular Biology, Herman Ostrow School of Dentistry, University of Southern California, Los Angeles, CA 90033, USA

**This PDF file includes:**

Supplementary Text

Figs. S1 to S6

Tables S1


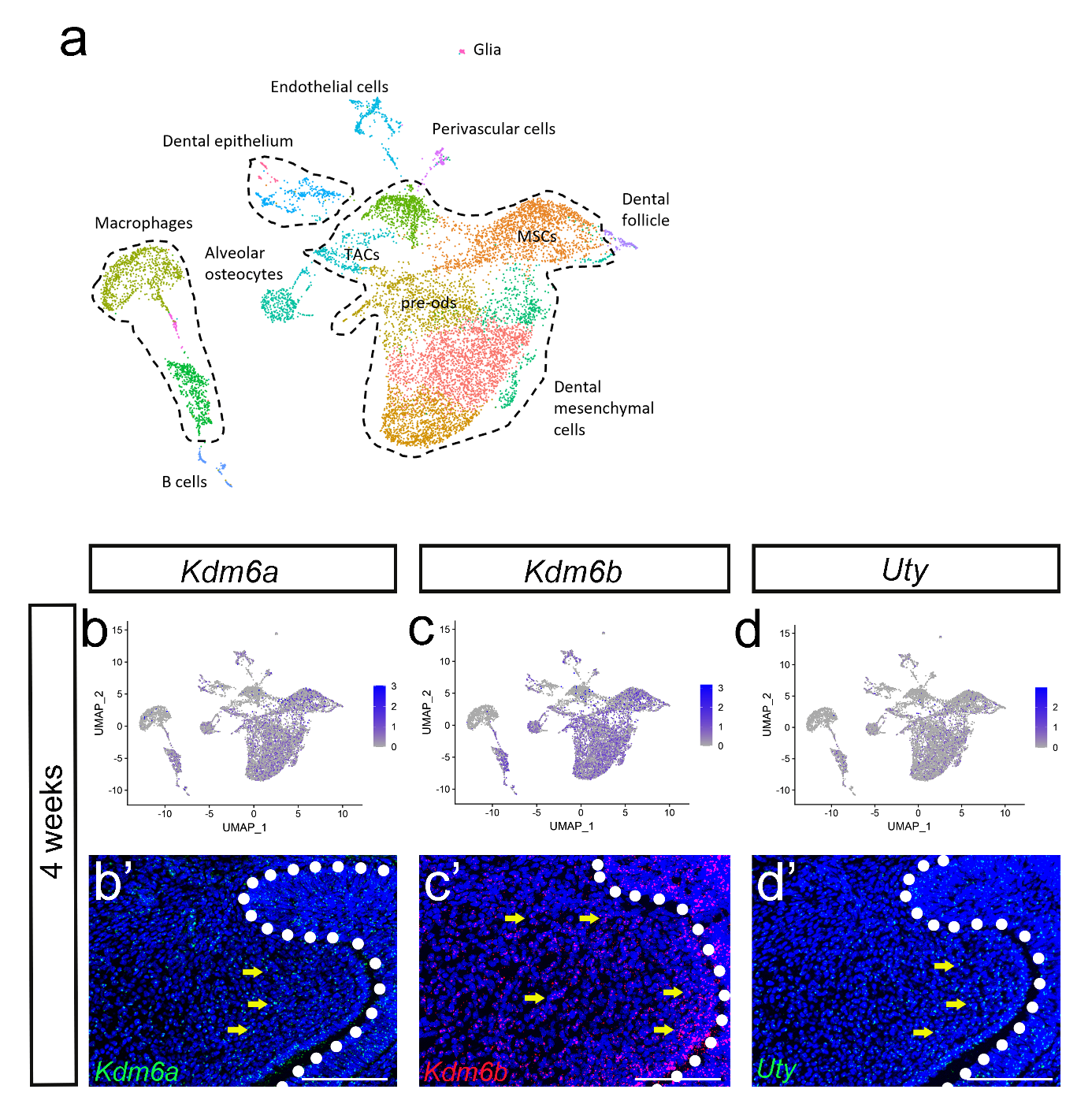


Fig. S1. KDM6 family expression patterns in the adult mouse incisor.

(a) UMAP plot of all cell types in wild-type mouse incisor samples. MSCs, mesenchymal stem cells; TACs, transit-amplifying cells; pre-ods, pre-odontontoblasts. (b, b’) Feature plot and *in situ* hybridization staining of *Kdm6a* in the wild-type mouse incisor. (c, c’) Feature plot and *in situ* hybridization staining of *Kdm6b* in the wild-type mouse incisor. (d, d’) Feature plot and *in situ* hybridization staining of *Uty* in the wild-type mouse incisor. White dotted lines outline the cervical loop. Yellow arrows indicate the positive cells. Scale bars: 50 µm.


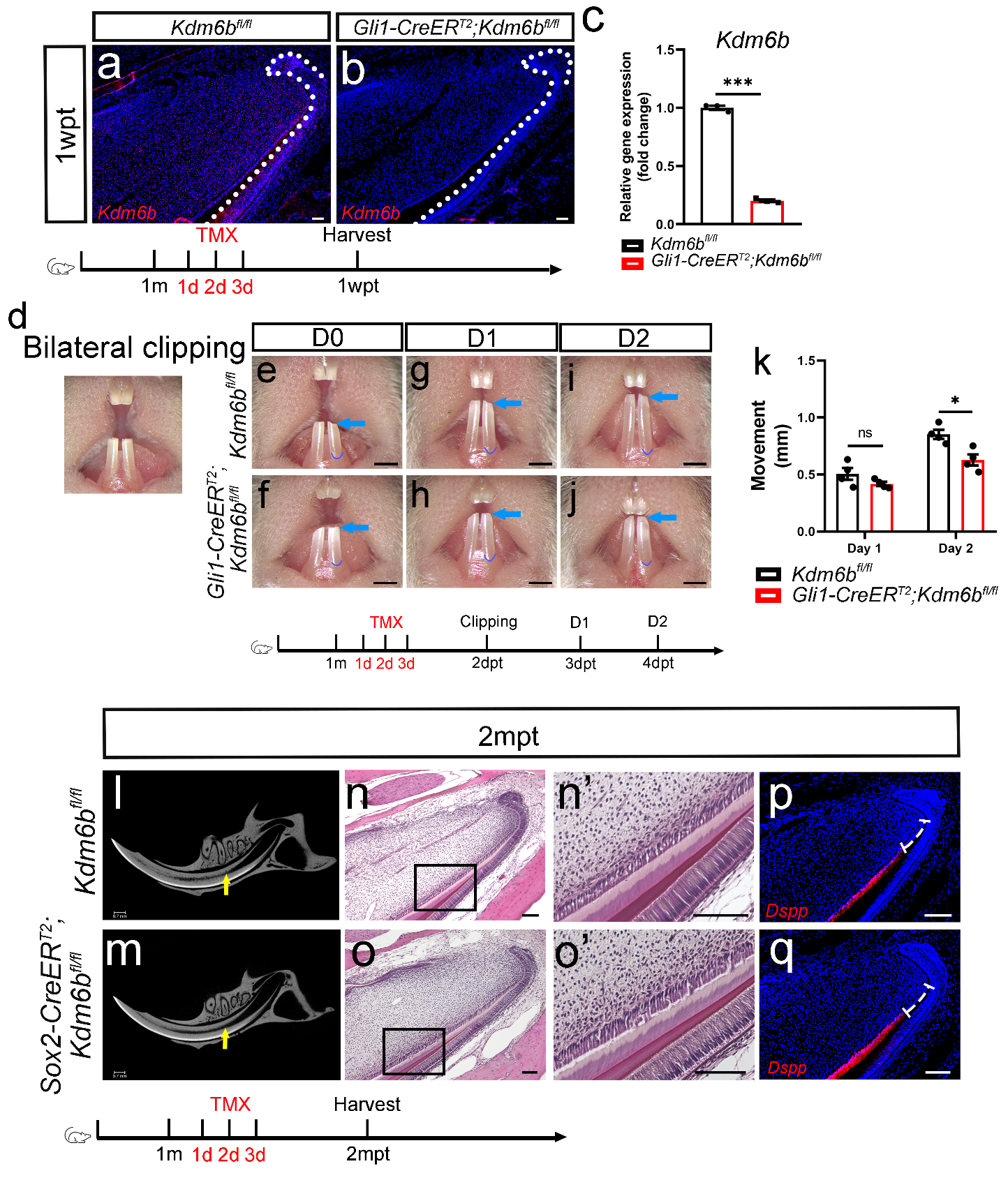


Fig. S2. KDM6B is essential for tissue homeostasis and incisor growth under mechanical loading in adult mice.

(a-c) *Kdm6b* expression in control and *Gli1-CreER^T2^;Kdm6b^fl/fl^* mouse incisors. (a-b) *In situ* hybridization staining of *Kdm6b* (red) in the incisors of control and *Gli1-CreER^T2^;Kdm6b^fl/fl^* mice. White dotted lines outline the cervical loop. Scale bars: 50 µm. (c) The mRNA expression of *Kdm6b* in control and *Gli1-CreER^T2^;Kdm6b^fl/fl^* mouse incisors. Data are represented as mean ± SEM. n = 3; *p* < 0.0001. (d-k) The incisor growth rate in the bilateral clipping model. (d) Schematic of the bilateral clipping model is shown below. (e-j) Representative images of clipped incisor growth in control and *Gli1-CreER^T2^;Kdm6b^fl/fl^* mice at different time points. Blue lines show the gingival margin. Blue arrows show the clipping position. Scale bars: 1 mm. (k) Quantiﬁcation of the incisor growth on day 1 and day 2. Data are represented as mean ± SEM. n = 4; *p* = 0.1645 (Day 1); *p* = 0.0116 (Day 2). (l-q) The phenotypes of control and *Sox2-CreER^T2^;Kdm6b^fl/fl^* mice at 2 mpt. (l-m) MicroCT of control and *Sox2-CreER^T2^;Kdm6b^fl/fl^* mouse incisors at 2 mpt. Yellow arrows indicate the dental pulp in (l-m). Scale bars: 0.7 mm. (n-o) HE staining of control and *Sox2-CreER^T2^;Kdm6b^fl/fl^* mouse incisors. Boxes in (n) and (o) are magnified in (n’) and (o’), respectively. Scale bars: 50 µm. (p-q) *Dspp* (red) *in situ* hybridization in control and *Sox2-CreER^T2^;Kdm6b^fl/fl^* mouse incisors. White dotted lines show the distance between cervical loop bending point and the odontoblast initiation. Scale bars: 50 µm.
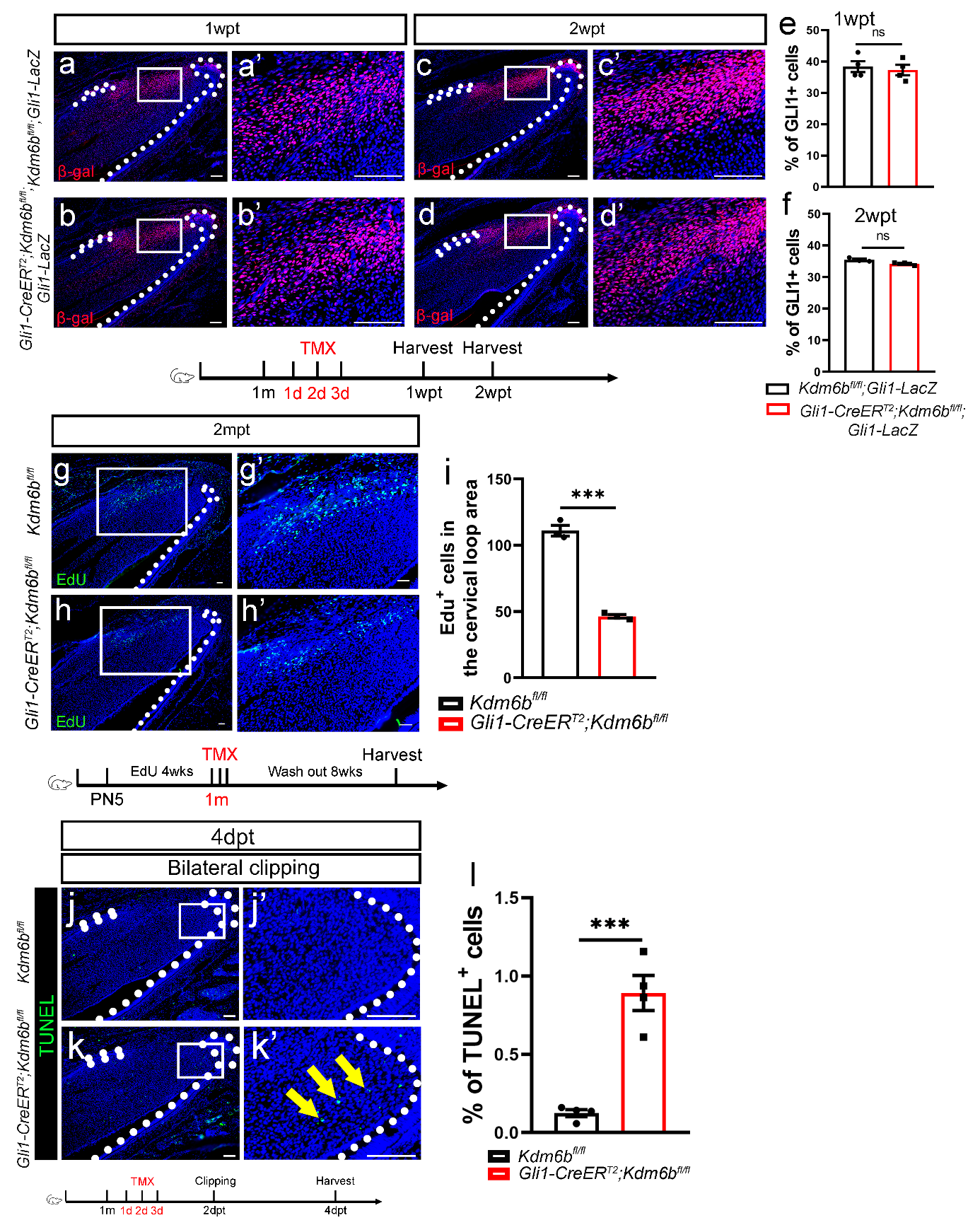


Fig. S3. KDM6B maintains TAC fate under mechanical loading.

(a-d) β-GAL immunofluorescence staining at 1 wpt (a-b) and 2 wpt (c-d) in *Gli1-LacZ* control and *Gli1-CreER^T2^;Kdm6b^fl/fl^;Gli1-LacZ* mice. (a’), (b’), (c’) and (d’) represent magnified images of the boxes in (a-d), respectively. White dotted lines outline the cervical loop. Scale bars: 50 µm. (e-f) Quantification of GLI1^+^ cells in dental mesenchyme at 1 wpt and 2 wpt. Data are represented as mean ± SEM. n = 3-4; *p* = 0.6768 (1 wpt); *p* = 0.0504 (2 wpt). (g-h) EdU staining (green) in the incisors of control and *Gli1-CreER^T2^;Kdm6b^fl/fl^* mice. (g’-h’) represent magnified images of the boxes in (g-h), respectively. White dotted lines outline the cervical loop. (i) Quantification of the EdU^+^ cells. Data are represented as mean ± SEM. n = 3; *p* = 0.0001. (j-l) TUNEL staining of bilateral clipped incisors (j-k) of control and *Gli1-CreER^T2^;Kdm6b^fl/fl^* mice at 4 dpt. (j’-k’) represent magnified images of the boxes in (j-k), respectively. White dotted lines outline the cervical loop. Yellow arrows indicate the positive cells. Scale bars: 50 µm. (l) Quantification of TUNEL^+^ cells in the dental mesenchymal region. Data are represented as mean ± SEM. n = 3; *p* = 0.0005.


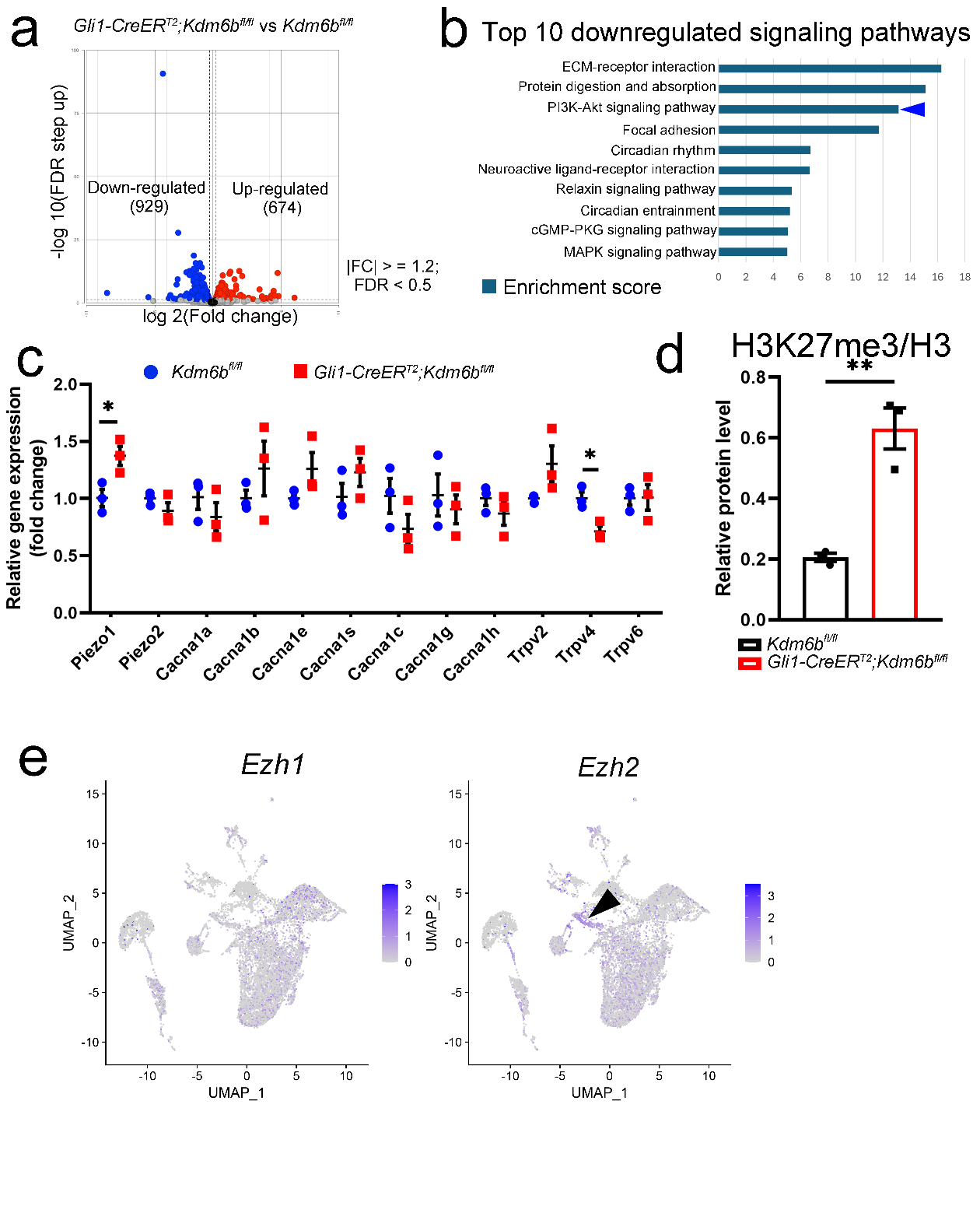


**Fig. S4.**

(a) Volcano plot of bulk RNA-seq data from the proximal region of control and *Gli1-CreER^T2^;Kdm6b^fl/fl^* mouse incisors at 5 days post-tamoxifen induction. (b) Top 10 downregulated signaling pathways identified by KEGG analysis using the differentially expressed genes from bulk RNA-seq data analysis. (c) Individual bar graph of the mRNA expression of the calcium signaling pathway-associated genes in control and *Gli1-CreER^T2^;Kdm6b^fl/^* mice. Data are represented as mean ± SEM. n = 3; *p* = 0.0315 (*Piezo1*); *p* = 0.2405 (*Piezo2*); *p* = 0.3519 (*Cacna1a*), *p* = 0.3576 (*Cacna1b*); *p* = 0.1567 (*Cacna1e*); *p* = 0.2762 (*Cacna1s*); *p* = 0.2193 (*Cacna1c*); *p* = 0.6016 (*Cacna1g*); *p* = 0.3357 (*Cacna1h*); *p* = 0.1283 (*Trpv2*); *p* = 0.0149 (*Trpv4*); *p* = 0.9601 (*Trpv6*). (d) Quantification of H3K27me3 and H3 protein levels in the proximal regions of control and *Gli1-CreER^T2^;Kdm6b^fl/fl^* mouse incisors. Data are represented as mean ± SEM. n = 3; *p* = 0.0035. (e) Feature plots of *Ezh1* and *Ezh2* in the wild-type mouse incisor. Black arrowhead indicates the positive cells.


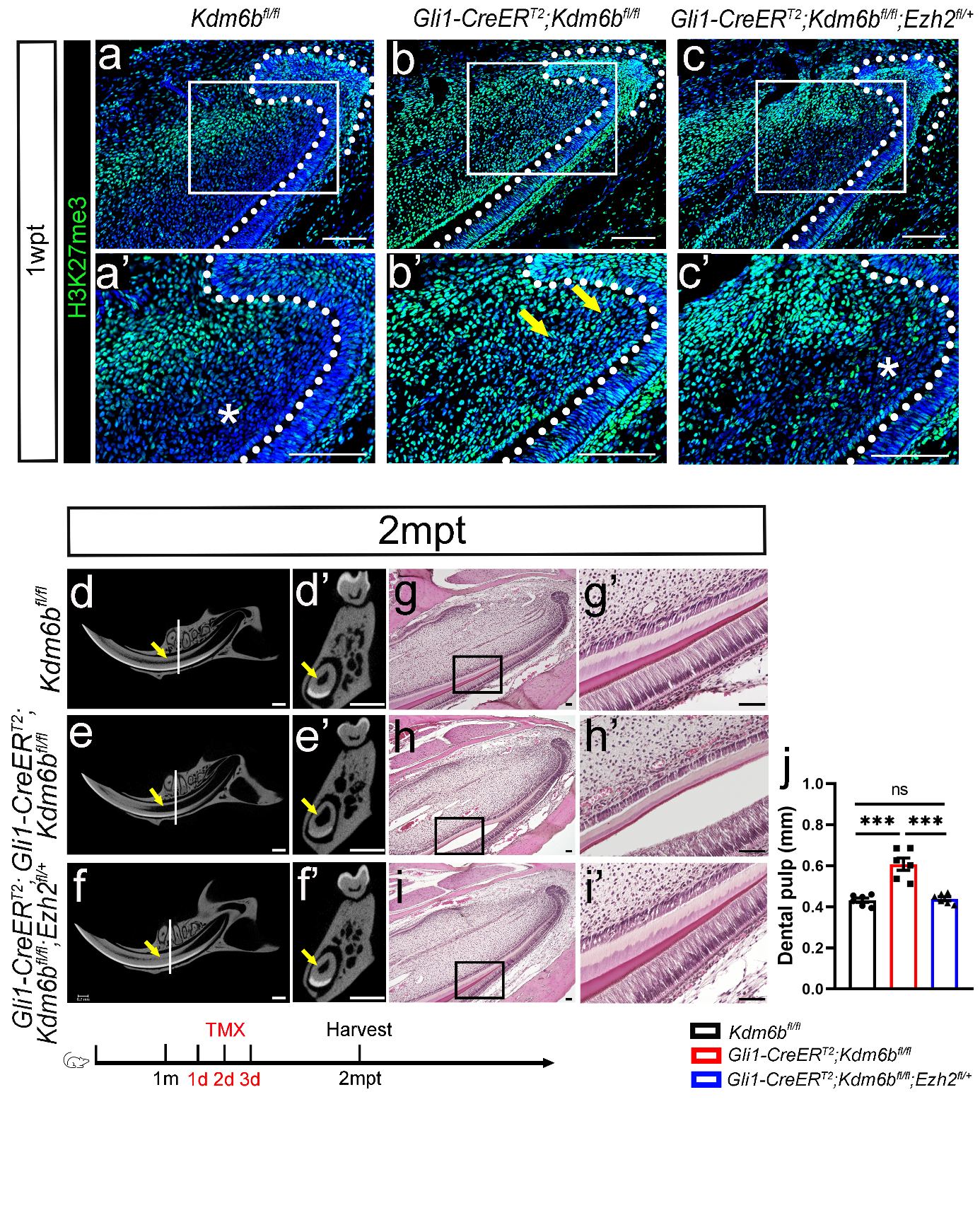


Fig. S5. The phenotype of *Gli1-CreER^T2^;Kdm6b^fl/fl^;Ezh2^fl/+^* mice.

(a-c) H3K27me3 immunofluorescence staining in control, *Gli1-CreER^T2^;Kdm6b^fl/fl^* , and *Gli1-CreER^T2^;Kdm6b^fl/fl^;Ezh2^fl/+^* mouse incisors. (a’-c’) represent magnified images of the boxes in (a-c). White dotted lines outline the cervical loop. Asterisk indicates the absence of signals. Scale bars: 50 µm. (d-i) The phenotypes of control, *Gli1-CreER^T2^;Kdm6b^fl/fl^* , and *Gli1-CreER^T2^;Kdm6b^fl/fl^;Ezh2^fl/+^* mice at 2 mpt. (d-f) MicroCT of control, *Gli1-CreER^T2^;Kdm6b^fl/fl^* , and *Gli1-CreER^T2^;Kdm6b^fl/fl^;Ezh2^fl/+^* mouse incisors at 2 mpt. (d’- f’) are coronal sections through the distal root of the first molar (white lines) in (d-f). Yellow arrows indicate the dental pulp in (d-f and d’- f’). Scale bars: 1 mm. (g-i) H&E staining of control, *Gli1-CreER^T2^;Kdm6b^fl/fl^* , and *Gli1-CreER^T2^;Kdm6b^fl/fl^;Ezh2^fl/+^* mouse incisors. (g’-i’) represent magnified images of the boxes in (g-i), respectively. Scale bars: 50 µm. (j) Quantiﬁcation of the diameter of the dental pulp cavities of control, *Gli1-CreER^T2^;Kdm6b^fl/fl^* , and *Gli1-CreER^T2^;Kdm6b^fl/fl^;Ezh2^fl/+^* mice. Data are represented as mean ± SEM. n = 6; *p* < 0.0001 (control vs *Gli1-CreER^T2^;Kdm6b^fl/fl^*); *p* < 0.0001 (*Gli1-CreER^T2^;Kdm6b^fl/fl^* vs *Gli1-CreER^T2^;Kdm6b^fl/fl^;Ezh2^fl/+^*); *p* = 0.9572 (control vs *Gli1-CreER^T2^;Kdm6b^fl/fl^;Ezh2^fl/+^*).


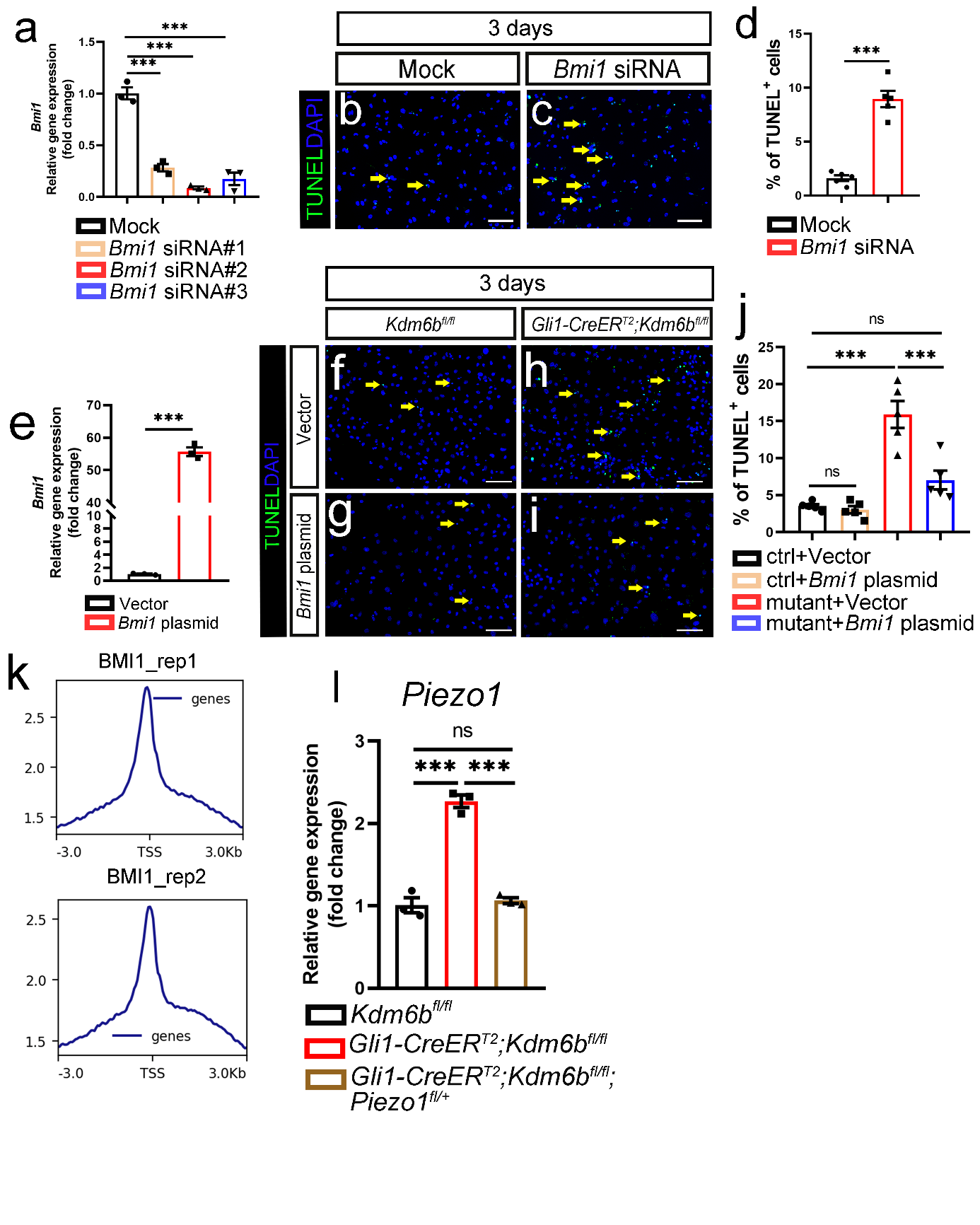


Fig. S6. *Bmi1* plays an essential role in cell fate and *Piezo1* expression after KDM6B loss.

(a) The mRNA expression of *Bmi1* in dental mesenchymal cells after 3 days of mock control or *Bmi1* siRNA treatment. Data are represented as mean ± SEM. n = 3; *p* < 0.0001 (siRNA 1); *p* < 0.0001 (*Bmi1* siRNA 2); *p* < 0.0001 (*Bmi1* siRNA 3). (b-c) TUNEL staining of dental mesenchymal cells from wild-type mouse incisors after 3 days of mock control or *Bmi1* siRNA treatment. Yellow arrows indicate the positive cells. Scale bars: 50 µm. (d) Quantification of TUNEL^+^ dental mesenchymal cells. Data are represented as mean ± SEM. n = 5; *p* < 0.0001. (e) The mRNA expression of *Bmi1* in dental mesenchymal cells after 3 days of vector or *Bmi1* plasmid treatment. Data are represented as mean ± SEM. n = 3; *p* < 0.0001. (f-i**)** TUNEL staining of the dental mesenchymal cells from control and *Gli1-CreER^T2^;Kdm6b^fl/fl^* mouse incisors after 3 days of vector or *Bmi1* plasmid treatment. Yellow arrows indicate the positive cells. Scale bars: 50 µm. (j) Quantification of TUNEL^+^ dental mesenchymal cells. Data are represented as mean ± SEM. n = 5; *p* = 0.9901 (ctrl+vector vs ctrl+*Bmi1* plasmid); *p* < 0.0001 (ctrl+vector vs mutant+vector); *p* = 0.1729 (ctrl+vector vs mutant+*Bmi1* plasmid); *p* = 0.0003 (mutant+vector vs mutant+*Bmi1* plasmid). (k**)** Plots of CUT&RUN sequencing data of BMI1 in the proximal region of wild-type mouse incisors. TSS: Transcription start sites. The sequence has two replications. (l) The mRNA levels of *Piezo1* in the proximal regions of control, *Gli1-CreER^T2^;Kdm6b^fl/fl^*, and *Gli1-CreER^T2^;Kdm6b^fl/fl^;Piezo1^fl/+^* mouse incisors. Data are represented as mean ± SEM. n = 3; *p* < 0.0001 (control vs *Gli1-CreER^T2^;Kdm6b^fl/fl^*); *p* < 0.0001 (*Gli1-CreER^T2^;Kdm6b^fl/fl^* vs *Gli1-CreER^T2^;Kdm6b^fl/fl^;Piezo1^fl/+^*); *p* = 0.8555 (control vs *Gli1-CreER^T2^;Kdm6b^fl/fl^;Piezo1^fl/+^*).

Table S1.Primers used for genotyping and qPCR.

| primers used for genotyping of mice | | |
| --- | --- | --- |
| Gene | Primer type | Primer sequence (5' to 3') |
| *Gli1-CreER^T2^* | Common | GGG ATC TGT GCC TGA AAC TG |
|  | Wild type Reverse | CTT GTG GTG GAG TCA TTG GA |
|  | Mutant Reverse | CAG GTT CTT GCG AAC CTC AT |
| *Sox2-CreERT2* | Internal Positive Control Forward | CTA GGC CAC AGA ATT GAA AGA TCT |
|  | Internal Positive Control Reverse | GTA GGT GGA AAT TCT AGC ATC ATC C |
|  | Transgene Forward | GCG GTC TGG CAG TAA AAA CTA TC |
|  | Transgene Reverse | GTG AAA CAG CAT TGC TGT CAC TT |
| *TdTomato* | Wild type Forward | AAG GGA GCT GCA GTG GAG TA |
|  | Wild type Reverse | CCG AAA ATC TGT GGG AAG TC |
|  | Mutant Reverse | GGC ATT AAA GCA GCG TAT CC |
|  | Mutant Forward | CTG TTC CTG TAC GGC ATG G |
| *Gli1-LacZ* | Common | GGG ATC TGT GCC TGA AAC TG |
|  | Mutant Reverse | TCT GCC AGT TTG AGG GGA CGA C |
|  | Wild type Reverse | AGG TGA GAC GAC TGC CAA GT |
| *Kdm6b^fl/fl^* | Forward | CAG CGA TCC TGA CTT GTT CA |
|  | Reverse | GTG CCA AGG CTG GAG GA |
| *Ezh2^fl/fl^* | Forward | CAT GTG CAG CTT TCT GTT CA |
|  | Reverse | CAC AGC CTT TCT GCT CAC TG |
| *Piezo1^fl/fl^* | Forward | GCC TAG ATT CAC CTG GCT TC |
|  | Reverse | GCT CTT AAC CAT TGA GCC ATC T |
| primers used for qPCR | | |
| *β-actin* | Forward | GGC TGT ATT CCC CTC CAT CG |
|  | Reverse | CCA GTT GGT AAC AAT GCC ATG T |
| *Kdm6b* | Forward | TGA AGA ACG TCA AGT CCA TTG TG |
|  | Reverse | TCC CGC TGT ACC TGA CAG T |
| *Piezo1* | Forward | TCATCATCCTTAACCACATGGTG |
|  | Reverse | TGAAGACGATAGCTGTCATCCA |
| *Piezo2* | Forward | AGAGTCGGAAAAGAGATACCCTC |
|  | Reverse | CCAGACGATACAGATGAGAAGGA |
| *Cacna1a* | Forward | CACCGAGTTTGGGAATAACTTCA |
|  | Reverse | ATTGTGCTCCGTGATTTGGAA |
| *Cacna1b* | Forward | AAGTGGCATCAAGGAGTCGC |
|  | Reverse | GCTAGGCGTGGCATAGAGG |
| *Cacna1e* | Forward | GATGGAGACTCGGACCAGAG |
|  | Reverse | TGACCGTGAAACAGTTCTGCC |
| *Cacna1s* | Forward | TCAGCATCGTGGAATGGAAAC |
|  | Reverse | GTTCAGAGTGTTGTTGTCATCCT |
| *Cacna1c* | Forward | ATGAAAACACGAGGATGTACGTT |
|  | Reverse | ACTGACGGTAGAGATGGTTGC |
| *Cacna1g* | Forward | TGTCTCCGCACGGTCTGTAA |
|  | Reverse | AGATACCCAAAGCGACCATCTT |
| *Cacna1h* | Forward | GGCACGAGGGAAGGATACTCT |
|  | Reverse | GTGACGAAGTAGACGGGGGA |
| *Trpv2* | Forward | TGCTGAGGTGAACAAAGGAAAG |
|  | Reverse | TCAAACCGATTTGGGTCCTGT |
| *Trpv4* | Forward | ATGGCAGATCCTGGTGATGG |
|  | Reverse | GGAACTTCATACGCAGGTTTGG |
| *Trpv6* | Forward | AGGGGTTAATACTCTGCCTATGG |
|  | Reverse | GCACCTCACATCCTTCAAACTT |
| gRNA for CRISPRi | | |
|  | PAM |  |
| *Bmi1* gRNA 1 | GGG | AACAGCGATCCCGACGAGGT |
| *Bmi1* gRNA 2 | TGG | ACATACCCTCATGAGCTTAG |
| *Bmi1* gRNA 3 | CGG | GCTGCTCCGGATTACTTGTT |
| *Piezo1* gRNA 1 | GGG | AGATACTCATCCCAGGCATA |
| *Piezo1* gRNA 2 | AGG | AGAGCTGGCTCAGTGATGAC |
